# Bacteria‐Derived 1‐Allylpiperazine Alleviates Symptoms of Parkinson's Disease Model by Promoting Autophagy

**DOI:** 10.1002/advs.78133

**Published:** 2026-10-08

**Authors:** Mingqin Qu, Xinyuan Zhang, Xinyi Chen, Jiaxin Shi, Meng Xu, Fuhao Wang, Jinling Lv, Jiaman Sun, Weiyi Niu, Zihan Liu, Li Li, Fufeng Liu, Xiangming Wang

**Affiliations:** ^1^ Department of Cell Biology School of Basic Medical Sciences Laboratory for Clinical Medicine Capital Medical University Beijing China; ^2^ Department of Anesthesiology Xuanwu Hospital Capital Medical University Beijing China; ^3^ Key Laboratory of Industrial Fermentation Microbiology of the Ministry of Education Tianjin Key Laboratory of Industrial Microbiology College of Biotechnology Tianjin University of Science and Technology Tianjin China; ^4^ Department of Neurology Inner Mongolia People's Hospital Hohhot China; ^5^ College of Sciences Tianjin University of Science and Technology Tianjin China

**Keywords:** 1‐Allylpiperazine, autophagy, mTOR, Parkinson's disease, probiotics

## Abstract

Parkinson's disease (PD) is a progressive neurodegenerative disorder with no available disease‐modifying therapies. Given their diverse health‐promoting properties, probiotics have emerged as a potential neuroprotective strategy. Through screening in transgenic *Caenorhabditis elegans* (*C. elegans*) models of α‐synuclein (α‐syn)‐induced PD, we identified the *Escherichia coli* (*E. coli*) strain E8 as a candidate that effectively suppressed dendritic degeneration and motor deficits. These neuroprotective improvements were attributed to the reduction of α‐syn aggregation, which was mechanistically associated with E8's ability to promote autophagy. Metabolomic profiling further pinpointed 1‐allylpiperazine (1‐ALPP) as a bioactive metabolite responsible for rescuing PD‐related impairments, which was sufficient to recapitulate the autophagy activation induced by E8. Subsequent mechanistic studies revealed that 1‐ALPP activated autophagy through the AMPK/mTOR/HLH‐30 pathway. Notably, 1‐ALPP effectively inhibited α‐syn fibrillogenesis and cytotoxicity in a dose‐dependent manner, potentially via the inhibition of α‐syn liquid‐liquid phase separation (LLPS). Molecular simulations demonstrated that 1‐ALPP binds to α‐syn, thereby preventing the critical conformational changes required for fibril formation. Strikingly, the protective effects of E8 against PD phenotypes were intergenerationally inherited, persisting for two generations in an *hpl‐2*‐dependent manner.

## Graphical abstract

Parkinson's disease currently has no disease‐modifying therapies. In *Caenorhabditis elegans*, *Escherichia coli* E8 alleviates α‐synuclein‐provoked PD‐related defects through its bioactive metabolite 1‐ALPP, which combats PD by triggering AMPK/mTOR/HLH‐30‐dependent autophagy and suppressing α‐synuclein LLPS‐mediated fibrillization.

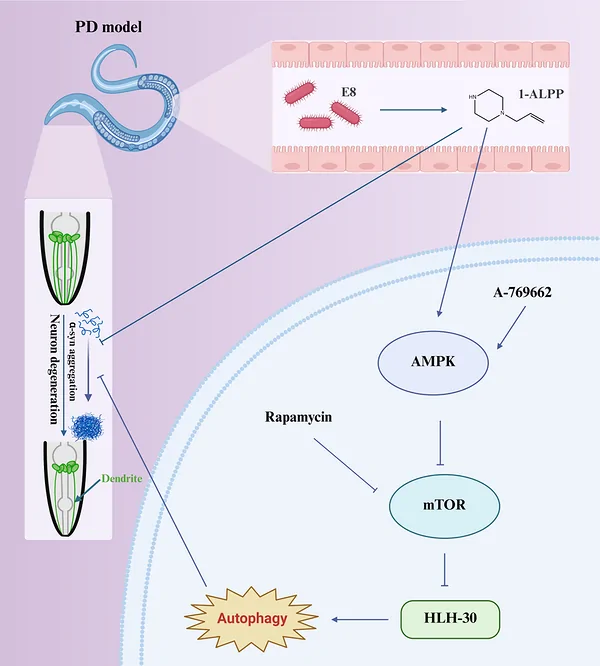

## 1 Introduction

PD is a chronic and progressively debilitating neurodegenerative disorder of the nervous system, primarily characterized by motor dysfunction and non‐motor symptoms [1, 2]. Its core pathological feature is the degeneration of dopaminergic neurons in the substantia nigra of the brain, which results in a significant reduction in dopamine levels, the neurotransmitter essential for coordinating movement [3, 4, 5]. Alpha‐synuclein (α‐syn), the primary structural component of Lewy bodies in PD, is strongly linked to familial PD due to pathogenic gene mutations. Under physiological conditions, α‐syn exists as a soluble monomer predominantly localized at presynaptic terminals in the central nervous system. However, under pathological conditions, it undergoes misfolding and aberrant aggregation, forming toxic oligomers and fibrillar structures. These aggregates ultimately mature into Lewy bodies and Lewy neurites, the hallmark of PD [6]. This abnormal aggregation is closely linked to various neurodegenerative disorders, with PD being the most representative. Current PD management prioritizes symptom control and quality‐of‐life enhancement, with clinical therapy predominantly centered on dopamine replacement. However, no existing interventions can effectively delay disease progression or fundamentally prevent its advancement and completely cure or halt of the disease remains unattainable.

In recent years, the emergence and advancement of the “microbiota‐gut‐brain axis” (MGBA) theory have highlighted the critical role of gut microbiota balance [7, 8, 9] and have sparked significant academic interest in exploring the potential of probiotics as a therapeutic strategy to modulate the pathological progression of PD [10]. Probiotics, renowned for their multifaceted health benefits, hold promise as a viable therapeutic option for PD. However, a vast array of microorganisms remains unexplored for their probiotic potential, necessitating an efficient screening system. Concurrently, the scarcity of effective PD drugs is partly attributed to the lack of rapid and high‐throughput screening models. *Caenorhabditis elegans*, with its simple structure, ease of cultivation, short life cycle, conserved genome, and amenability to genetic manipulation, is exceptionally well‐suited for large‐scale and rapid screening of potential probiotic candidates and drug discovery [11]. Additionally, *C. elegans* possesses a gut microbiota akin to that of humans [12], making it an ideal model for studying the effects of individual probiotics on PD. Laboratories can conveniently cultivate single bacterial species, facilitating the exploration of specific probiotic effects on PD models. Multiple PD models have been established using *C. elegans* as a model organism, which have been instrumental in mechanism research, gene screening, and other related studies [13]. It could also be used to identify bacterial genes that promote host neurodegeneration [14].

Although progress has been made in exploring probiotics for alleviating PD, most existing research has predominantly focused on a limited number of bacterial species‐*Bifidobacterium*, *Lactobacillus*, and *Bacillus subtilis*, while the mechanisms underlying their therapeutic effects remain incompletely elucidated [15, 16, 17]. It is noteworthy that microbial diversity in nature is vast, and many potential probiotics, which have yet to be thoroughly investigated, may exert therapeutic effects through diverse pathways, such as regulating the MGBA, generating bioactive metabolites, or modulating immune responses. This area urgently requires systematic exploration to uncover their full therapeutic potential.

In this study, we performed an unbiased screening of multiple bacteria using a transgenic *C. elegans* PD model. Among these, an *Escherichia coli* isolate E8 emerged as a protective bacterium against PD‐related dopaminergic dendritic degeneration. Mechanistic studies revealed that E8 likely exerts its neuroprotective effects by enhancing autophagy. Metabolomic analysis coupled with targeted screening identified 1‐ALPP as a metabolite responsible for alleviating PD symptoms through enhancement of autophagy. Intriguingly, the neuroprotective effects of E8 were heritable across two subsequent generations of *C. elegans*, with this intergenerational protection mediated by *hpl‐2*.

## 2 Methods

### 2.1 *C. elegans* Strains and Culture

All *C. elegans* strains were maintained at 20°C under standard conditions on nematode growth medium (NGM) plates seeded with *E. coli* OP50 [18], unless otherwise specified. The wild‐type strain N2 served as the genetic background for all other strains used in this study. A complete list of the strains employed is provided below.

**Table:** 

Strains	Genotype
	A30P [P*dat‐1*::α‐syn (A30P); P*cat‐1*::EYFP]
	P*cat‐1*::EYFP
	P*dat‐1*::α‐syn (A30P)
BY250	*vtIs7* [P*dat‐1*::GFP]
DA2123	*adIs2122* [P*lgg‐1*::GFP::LGG‐1* + rol‐6*(*su1006*)]
MAH215	*sqIs11* [P*lgg‐1*::mCherry::GFP::LGG‐1; *rol‐6*]
NL5901	*pkIs2386* [P*unc‐54*::α‐syn::YFP; *unc‐119*(+)]
TYL001	*syuIs1* [P*cpr‐5*::GFP; *rol‐6*(+)]
XMW128	A30P; *sid‐1*
XMW130	A30P; *hlh‐30*
XMW221	P*dat‐1*::α‐syn (A30P)::GFP; P*dat‐1*::mCherry
XMW224	A30P; *pek‐1*
XMW230	A30P; *set‐18*
XMW234	A30P; *pink‐1*
XMW235	A30P; *epg‐6*
XMW237	A30P; *aak‐2*
XMW239	A30P; *hpl‐2*
XMW245	A30P; *atg‐13*
*bpIs151*	P*sqst‐1*::SQST‐1::GFP; *unc‐76*(+)

### 2.2 Bacterial Strains and Growth Conditions

The bacterial strains employed in this study encompassed environmental isolates (E2, E4, E5, E7, E8, E9, E10, E11‐1, E11‐2, E14‐1, E14‐2, E15‐1, E19, E20, E21, E22) and strains isolated from fermented vegetables (FV1, FV4, FV5). The bacterial strains in the environment were obtained during routine laboratory maintenance. Non‐OP50 colonies were observed on the NGM plates; these colonies were subsequently inoculated by streaking onto fresh NGM agar plates to obtain individual isolates. The bacterial strains from fermented vegetable samples were obtained by placing the fermented vegetables in sterile microcentrifuge tubes containing sterile distilled water, and then streaking the resulting suspension onto NGM agar plates to separate them. These strains were streaked individually onto NGM agar plates and incubated at 28°C for 24 h to obtain single colonies. A single colony was then inoculated into 1 mL of TSB (Hopebio, Cat. HB4114) liquid medium, which was shaken at 28°C for 24 h to generate a bacterial suspension. Laboratory *E. coli* strains (OP50, DH5α, trans5α, top10, trans10, HT115) were incubated at 37°C for 24 h to obtain single colonies; then the single colony was inoculated into 1 mL of LB (OXOID, Cat. LP0021B) liquid medium, which was shaken at 37°C for 24 h. Subsequently, 60 µL of the bacterial suspension was spread onto NGM plates and cultured at the corresponding temperature for one day prior to experimentation. Glycerol stocks of all strains were stored at −80°C.

### 2.3 Quantification of the Number of Dendrites

As for A30P worms, we conducted a quantitative analysis of the six dendritic branches located between the first pharyngeal bulb and the tip of the nose—these branches originate from four CEP neurons and two serotonergic ADF neurons. The dendritic projections of these six neurons are distributed across multiple focal planes in three‐dimensional space. During the quantitative process, we carefully adjusted the focus knobs to scan all the focal planes and counted each dendrite originating from these six neurons. Representative images show a single focal plane.

### 2.4 Quantification of α‐syn Aggregation in *C. elegans*

After washing L4‐stage α‐syn worms five times with M9 buffer, transfer them to NGM plates seeded with OP50 or E8 (3 worms per group). After 3 days, transfer L4‐stage progeny to fresh plates containing OP50 or E8 for further cultivation. On day 5 after L4, observe under a fluorescence microscope and count α‐syn protein aggregates (puncta >1 µm^2^) in the head region.

### 2.5 Quantification of α‐syn Aggregates in Dopaminergic Neurons

After washing the L4‐stage P*dat‐1*::α‐syn (A30P)::GFP; P*dat‐1*::mCherry worms with M9 buffer, they were transferred to NGM culture plates inoculated with OP50 or E8. Three days later, the L4‐stage offspring were transferred to new culture plates containing OP50 or E8 for further cultivation. On the second day after the L4 stage, the worms were observed under a fluorescence microscope, and the number of green aggregation points in the dendrites was counted.

### 2.6 Exposure of *C. elegans* to 6‐OHDA

The P*dat‐1*::GFP (BY250) worms were synchronized to obtain L3‐stage worms. After washing with M9 solution, they were added to the following mixed solutions: 50 mM 6‐hydroxydopamine (6‐OHDA, Orileaf, Cat. S88807) + 10 mM ascorbic acid (AA, Aladdin, Cat. A103534) solution, 50 mM 6‐OHDA + 10 mM AA + 2 mM levodopa (Orileaf, Cat. B21710) solution, 50 mM 6‐OHDA + 10 mM AA + E8 bacterial solution. The worms were incubated at 20°C for 1 h and gently mixed once every 15 min. Then, the worms were washed with M9 solution and placed on NGM OP50 or OP50 + 2 mM levodopa or E8 plates. 72 h after exposure to 6‐OHDA, the number of normal (non‐fractured) dopaminergic neurons’ dendrites from the first pharyngeal bulb to the nose in the worm's head was observed and counted using a fluorescence microscope at different focal planes.

### 2.7 16S rRNA Gene Sequencing

The E8 monoclonal culture was inoculated into TSB medium and incubated at 28°C with shaking for 24 h. After centrifugation, the supernatant was discarded. A portion of the bacterial pellet was transferred to 20 µL lysis buffer. The 10× lysis buffer solution was prepared by mixing 25 mL of 0.5 M KCl, 5 mL of 10 mM disodium EDTA, 5 mL of 250 mM Tris, and 15 mL of ddH_2_O. For the 2× working solution, 200 µL of 10× lysis solution was combined with 10 µL of Tween‐20, 10 µL of proteinase K, and 780 µL of ultrapure water. Lysis was performed in a PCR thermal cycler at 65°C for 70 min, followed by 95°C for 15 min. PCR amplification was carried out using the universal bacterial 16S rRNA primers 341_F (5′‐ACTCCTACGGGAGGCAGCAG‐3′) and 1492_R (5′‐GGTTACCTTGTTACGACTT‐3′). The 20 µL PCR reaction mixture contained 10 µL of 2× Taq PCR MasterMix, 1 µL of each primer, 1 µL of bacterial genomic DNA template, and 7 µL of nuclease‐free water. The amplification conditions were as follows: initial denaturation at 95°C for 5 min; 38 cycles of 95°C for 30 s, 55°C for 30 s, and 72°C for 2 min; and a final extension at 72°C for 10 min. The PCR products were analyzed by 2% agarose gel electrophoresis, sequenced, and the obtained 16S rRNA gene sequences were compared with the NCBI core nucleotide database using the nucleotide BLAST alignment tool.

### 2.8 Whole Genome Sequencing

The E8 monoclonal strain was inoculated into TSB medium and cultured in a shaker at 28°C overnight. Then, the culture was centrifuged at 12 000 rpm for 10 min at 4°C; the supernatant was discarded, and the bacteria were collected and sent to BGI for sequencing. First, genomic DNA was extracted. The purified genomic DNA was quantified, and high‐quality DNA was used for the subsequent library construction and sequencing. Then, Illumina library construction and genomic sequencing were carried out. Genomic DNA fragmentation was performed using Covaris, and the DNA samples were fragmented into fragments of approximately 350 bp. The library was prepared using the NEXTFLEX Rapid DNA‐Seq Kit. Then, the data generated by the Illumina platform were used for bioinformatics analysis. The predicted CDS were annotated in NR, Swiss‐Prot, Pfam, GO, COG, KEGG, CAZY, etc. databases using sequence comparison tools such as BLASTP, Diamond, and HMMER to obtain the functional information of the genes. The secondary metabolic product synthesis gene clusters were predicted using antiSMASHv5.1.2.

### 2.9 CFU Assay

To prepare a bacterial suspension, select one E8 monoclonal colony and culture it in 1 mL of TSB solution at 28°C for 24 h. Perform serial dilutions by mixing 1 mL of the bacterial suspension with ddH_2_O at 10^6^, 10^7^, 10^8^, and 10^9^ dilution factors. Plate 100 µL from each dilution onto NGM plates, spread evenly, and incubate at 28°C for 24 h. The following day, count the colonies on plates with about 30–250 colonies and calculate the colony‐forming units (CFU) per mL of the original bacterial suspension.

### 2.10 Basal Slowing Assay

The basal slowing assay was conducted as previously described [19, 20]. Transfer 5 L4‐stage P*dat‐1*::A30P worms onto NGM plates seeded with either *E. coli* OP50 or E8/1‐ALPP and incubate at 20°C for 3 days. Subsequently, transfer L4‐stage F1 offspring to fresh NGM plates coated with the corresponding bacterial strain and incubate for an additional 1 day at 20°C. For the assay, wash D1‐stage P*dat‐1*::A30P worms three times with M9 buffer, then transfer them to NGM plates with or without a lawn of *E. coli* OP50 (with an inner diameter of ∼1 cm and an outer diameter of ∼5 cm) and allow them to recover for 90 s. Subsequently, record the number of body bends within 20 s. When the entire head region of the worm shifts from one lateral side to the contralateral side, the bending is deemed to have completed one cycle. The slowing rate is calculated by the following formula: Slowing rate = (N_without food_−N_with food_)/N_without food_.

### 2.11 Food Choice Assay

Dilute the OP50 bacterial culture with ultrapure water to achieve a final concentration of 11.33 × 10^8^ CFU/mL for both OP50 and E8. Prepare a fresh, sterile 35 mm NGM plate by drawing a central line and marking the 1/4 and 3/4 positions. Spot 4 µL of the diluted OP50 and E8 bacterial suspensions onto the corresponding marked positions. After washing 50 L4‐stage P*dat‐1*::A30P or N2 worms 5 times with M9 buffer, transfer them to the midpoint of the line. As each worm reaches either bacterial spot, immediately remove it and record the number of worms that choose OP50 versus E8.

### 2.12 Fecundity Assay

Prepare three NGM plates, each coated with either *E. coli* OP50 or E8. For each plate, transfer a single L4‐stage P*dat‐1*::A30P worm that has been washed with M9 buffer. Every 24 h, transfer the worm to a fresh corresponding NGM plate and count the number of eggs laid on the previous plate. Continue this process until egg‐laying ceases, then calculate the total number of eggs produced by each worm.

### 2.13 Growth Rate Assay

To assess bacterial strain effects on development, perform the following procedure: First, clean L4‐stage P*dat‐1*::A30P worms in M9 buffer and transfer 3 worms each onto OP50 and E8 NGM plates. After 3 days, select 6 L4‐stage offspring from each plate and transfer them to fresh corresponding bacterial plates. 24 h later (designated as Day 1, D1), transfer the worms to new plates. Three hours post‐transfer, remove all adult worms, leaving only eggs on the plates. After three days of incubation, quantify the number of worms that have reached the L4 stage versus total worms.

### 2.14 Motor Ability Assay

To assess worm motility, perform the following procedure: Pipette 200 µL of M9 buffer onto a clean glass slide. Using a platinum wire pick, carefully transfer a single worm to the M9 solution. Allow a 30‐s period for the worm to acclimate to the new environment. Following the adaptation period, quantify the number of complete body bends (swings) executed by the worm within a 30‐s observation window. A single swing is defined as a full sinusoidal wave motion involving coordinated movement of both the head and tail in the same direction. Partial movements restricted to either the head or tail region alone should not be counted as complete swings. Record the total number of complete swings observed during the measurement period. During the behavioral assessment and counting process of this study, we did not employ single‐blind methods. This is one of the limitations of our study. However, to minimize potential observer bias and ensure the reliability of the results, all experiments were independently repeated by at least two researchers.

### 2.15 Body Bends Assay

Five L4‐stage N2 or α‐syn worms were transferred to NGM plates inoculated with *E. coli* OP50 or E8/1‐ALPP. They were cultured at 20°C for 3 days. Subsequently, the L4‐stage F1 offspring were transferred to fresh NGM plates coated with the corresponding strains. The N2 group and the α‐syn group were further cultured at 20°C for another 5 days, and then were placed on the NGM plates to freely move for 30 s, and the number of body bends within 20 s was recorded.

### 2.16 Heat Stress Assay

To assess heat stress resistance, perform the following procedure: Inoculate three N2 and α‐syn worms each onto OP50 and E8 NGM plates, respectively. After three days of incubation, transfer ≥ 20 L4‐stage offspring from each strain to fresh corresponding bacterial plates. On Day 1 (D1), subject the N2 worms to 35°C heat stress for 4 h, while subject the α‐syn worms to 35°C heat stress for 150 min. Following the heat stress treatment, monitor and record the survival rate of worms in each group.

### 2.17 Intestinal Colonization

To assess bacterial colonization in the *C. elegans* intestine, three P*dat‐1*::A30P L4‐stage worms were washed 5 times with M9 buffer and individually transferred to plates seeded with either OP50 or E8. After reproduction, 80–90 L4‐stage offspring from each bacterial strain were transferred to fresh OP50‐ or E8‐coated plates. At days 1 (D1), 3 (D3), and 5 (D5) post‐transfer, 20 worms per condition were randomly selected and anesthetized for microscopic examination. Using 40× differential interference contrast (DIC) microscopy, intestinal colonization was determined by observing bacteria in the intestinal lumen. A worm was considered colonized if the intestinal cavity appeared with clearly discernible bacteria.

### 2.18 Lifespan Assay

To assess lifespan, L4‐stage offspring were washed 5 times with M9 buffer and transferred to fresh NGM plates seeded with either *E. coli* strain E8 or OP50 (designated as Day 0). Worms were transferred to new plates coated with the corresponding bacterial strain every 2 days to ensure consistent food availability. Mortality was recorded daily by gently prodding each worm for 5 s; the absence of a response was scored as death. The experiment continued until all worms on the plate had died [21].

### 2.19 E8 Functional Part Assay

OP50 and E8 monoclonal cultures were separately inoculated into 15 mL of TSB medium and incubated at 28°C for 24 h with shaking. The cultures were then centrifuged at 12 000 rpm for 10 min at 20°C. The supernatant was filtered and mixed with OP50 for plating. The resulting pellet was washed twice with ddH_2_O and resuspended in 500 µL ddH_2_O. One aliquot was subjected to sonication for 5 min, then filtered, while another was heat‐treated at 70°C for 30 min. A third aliquot was autoclaved at 121°C for 20 min. All treated samples were resuspended in 500 µL ddH_2_O and mixed with OP50 for subsequent plating.

### 2.20 Fluorescence Microscopy and Visualization Assay

A 3% agarose solution was prepared, microwaved, and dropped onto a slide, then pressed with a coverslip to form circular gaskets. 2.5 µL of 4 mg/mL levamisole in M9 was transferred to a gasket. Worms were immersed in the anesthetic using a platinum wire and imaged on a Zeiss Imager M2 microscope with filter sets 20 (Rhodamine) and 38 (Endow GFP). The dendrites of dopaminergic neurons were observed at 40× magnification, and the number of dendrites was counted. The cell bodies of P*dat‐1*::GFP head neurons were captured under a 10× magnification, and the fluorescence intensity was quantified. Under a 10× magnification, the number of autophagy substrates in the SQST‐1::GFP head was counted.

### 2.21 Transcriptome Analysis

Four L4‐stage A30P worms were inoculated onto six plates, each seeded with either E8 or OP50 *E. coli*. After 5 days of incubation, the worms were rinsed with sterile M9 solution and collected in 1.5 mL microcentrifuge tubes. Following natural sedimentation, the supernatant was carefully aspirated, and the worms were washed an additional five times with M9 solution to ensure thorough removal of residual bacteria. The washed worm pellets were then stored at −80°C for subsequent analysis. This entire procedure was performed in triplicate to ensure biological reproducibility. The TRIzol method was used for nucleic acid extraction. The extracted nucleic acids were quantified by Nanodrop2000, and their integrity was assessed by Agilent 2100 or LabChip GX. The library construction process utilized the VAHTS Universal V6 RNA‐seq Library Preparation Kit (Cat. NRM604‐01), which involved two rounds of mRNA binding and purification on magnetic beads (Cat. N411‐03), high‐temperature denaturation, fragmentation, and subsequent synthesis of the first and second strands of cDNA, followed by purification of the double‐stranded products. Then, end repair, 3′ end A‐tailing, and adapter ligation were performed. The ligation products were purified by magnetic beads and subjected to fragment selection before PCR amplification. The final libraries were quality‐checked by Qsep‐400, and those that passed were used for sequencing. During the sequencing stage, the DNBSEQ‐T7 platform from BGI was employed. DNA nanospheres were formed through circularization and rolling circle amplification, and the sequence reads were obtained using the combined probe anchor polymerization technology, yielding the raw sequencing data. The transcriptome sequencing and subsequent data analysis were carried out by Beijing Tsingke Biotechnology Co., Ltd.

### 2.22 Metabolome Analysis

A bacterial colony of OP50 or E8 was inoculated into 15 mL of TSB medium and cultured at 28°C for 24 h. The bacterial culture was centrifuged at 12 000 rpm for 10 min, and the supernatant was discarded. The centrifugation step was repeated under the same conditions. The bacterial precipitate obtained was resuspended and washed with ultrapure water, then subjected to another round of centrifugation. After discarding the supernatant, the precipitate was resuspended in ultrapure water and centrifuged three more times under the same conditions. Finally, the bacterial precipitate was collected and stored at −80°C. The above experiment was independently repeated to collect three samples. All the collected bacterial samples were sent to Tsingke Biotechnology (Beijing) Co., Ltd. for non‐targeted metabolomics analysis. The separation was carried out using an ultra‐high‐performance liquid chromatography system (Thermo Vanquish) in combination with a Waters BEH Amide chromatographic column. The mobile phase consisted of an aqueous phase containing 25 mmol/L acetic acid ammonium and ammonia water, as well as acetonitrile. Mass spectrometry was performed on the Orbitrap Exploris 120 instrument, with alternating acquisition of positive and negative ion modes, and the resolution was 60 000 (full scan) and 15 000 (secondary spectra). The raw data were converted to mzXML format using ProteoWizard and analyzed for peak extraction, qualitative and quantitative determination through an independently developed R program package.

### 2.23 Drug Treatment

Bacterial metabolite stocks were prepared and diluted to the desired working concentrations. For instance, 1‐ALPP (LeYan, Cat. 1202888, purity: 99.69%) was diluted in an OP50 bacterial suspension to achieve a final concentration of 1%. To prepare the experimental plates, 60 µL of the diluted metabolite‐bacterial mixture was spread evenly onto NGM plates. In all experiments, an OP50 bacterial culture plate without any added compounds served as the untreated control. Rapamycin (Innochem, Cat. A95804) was dissolved in DMSO to prepare a 100 mM storage solution. 3‐Methyladenine (Targetmol, Cat. T1879) was dissolved in water to a 10 mM storage solution. MG‐132 (Orileaf, Cat. S42096) was dissolved in DMSO to a 50 mM storage solution. A‐769662 (TCI, Cat. H1552) was dissolved in DMSO to a 100 mM storage solution. Dilute each storage solution with OP50 and then spread it on the NGM plate.

### 2.24 Real‐Time Quantitative PCR (RT‐qPCR)

The A30P worms were treated with OP50 or 1‐ALPP and collected. RNA was extracted using the Ultrapure RNA Kit (CWBio, Cat. CW05181S), and cDNA was obtained using the reverse transcription reaction kit (CWBio, Cat. CW3371M). Three replicate real‐time PCR reactions were performed for each cDNA sample, using the SuperStar Universal SYBR Master Mix solution (CWBio, Cat. CW3360M). The gene‐specific primers are listed in the table below. The gene expression levels were determined using the CFX96TM Real‐Time System (Bio‐Rad, USA) and normalized with *act‐1* as the internal reference.

**Table:** 

Gene name	Prime sequences (5′−3′)
*hlh‐30*	ATGTCGATGGCAAGACCGATTC
	AATGTCCTTCTTCCTGCGGTC
*atg‐13*	CTACAGTCGGCATACAATCCAG
	CTCATCGGAGAACGAATTGACG
*lgg‐1*	ACCCAGACCGTATTCCAGTG
	TTCGACCTCTCCTCCATACAC
*act‐1*	ACGAGGCCCAATCCAAGAG
	CTTCAGTGAGGAGGACTGGG

### 2.25 Preparation of Recombinant α‐synuclein

Recombinant α‐syn was produced in *E. coli* BL21 cells at 37°C under shaking conditions as described before [22]. 0.5 mM IPTG (Solarbio, Cat. I8070) was introduced when the optical density of cells was between 0.6 and 0.8. The cells were stimulated to express α‐syn for 4 h at 37°C. Following harvesting, a Ni‐NTA affinity column (Solarbio, Cat. P2010) was used to purify the target protein. Finally, the obtained recombinant protein was lyophilized and kept at −80°C for further use.

### 2.26 ThT Fluorescence Assay

The lyophilized powder of α‐syn was resuspended to a final concentration of 50 µM as described before [23]. To get rid of produced aggregates, the solution was centrifuged at 14 000 rpm after being sonicated for 15 min at 4°C. As a stock solution, 10 mM of 1‐ALPP was dissolved in 20 mM TBS buffer. In this study, a ThT (Macklin, Cat. T787366) fluorescence assay was used to measure the occurrence and development of α‐syn fibrils. In a 96‐well plate, 200 µL of α‐syn (50 µM) solution and 20 µL of ThT (250 µM) solution were co‐incubated with or without different concentrations of 1‐ALPP at 37°C. Lastly, each sample's fluorescence intensity was determined using the microplate reader (TECAN Infinite 200PRO, Austria) with an excitation wavelength of 440 nm and an emission wavelength of 480 nm.

### 2.27 Cytotoxicity Test

PC12 cells were cultured in RPMI 1640 (Gibco, Cat. 11875093) medium (containing 1% penicillin–streptomycin (Innochem, Cat. B46667) and 10% fetal bovine serum (NEWZERUM, Cat. FBS‐CE500)) in a 37°C, 5% CO2 incubator. Prior to cytotoxicity assays, cells were counted and seeded at approximately 1 × 10^4^ cells per well in a 96‐well plate during the logarithmic growth phase. RPMI 1640 medium was added to achieve a total volume of 100 µL per well. After 24 h, cells adhered to the plate. In the assessment of 1‐ALPP‐induced cytotoxicity, the experimental group was treated with different concentrations of 1‐ALPP, while the control group received an equal volume of PBS, followed by incubation for 24 h. To evaluate the protective effect of 1‐ALPP against α‐syn‐induced toxicity, the experimental group was treated with 25 µM mature α‐syn together with different concentrations of 1‐ALPP, whereas the control group received an equal volume of PBS, followed by incubation for 24 h [24]. After incubation, 10 µL of CCK‐8 (Solarbio, Cat. CA1210) solution was added to each well, taking care to avoid introducing bubbles. The wells were incubated at 37°C for 2 h, after which absorbance was measured at 450 nm using a TECAN Infinite 200 PRO (Austria) microplate reader.

### 2.28 Molecular Dynamics Simulation

3D structure of the α‐syn pentamer was obtained from the Protein Data Bank (PDB ID: 6H6B), and the chemical structure of 1‐ALPP was retrieved from the PubChem database (CAS: 13961‐36‐9) [25]. The topology file of 1‐ALPP was generated using AmberTools and ACPYPE. All MD simulations were performed using GROMACS 2022.6 with the Amber14SB force field [26]. The 1‐ALPP–α‐syn pentamer complex system was constructed by randomly inserting 10 1‐ALPP into a cubic simulation box (9.58377 nm × 9.58377 nm × 9.58377 nm). The minimum distance between the α‐syn pentamer and the box boundary was set to 1.2 nm. The system was solved with TIP3P water, and 45 chloride ions were added to neutralize the charge. Energy minimization was carried out using the steepest descent and conjugate gradient algorithms, followed by 100 ps of NVT and 100 ps of NPT equilibration with initial velocities assigned according to the Maxwell distribution. Finally, 100 ns production simulations were performed for both the α‐syn system and the 1‐ALPP–α‐syn system. Following the molecular dynamics simulation, root mean square deviation (RMSD), root mean square fluctuation (RMSF), and solvent accessible surface area (SASA) analyses were performed using the built‐in plugins of GROMACS of gmx rms, gmx rmsf, and gmx sasa, respectively. The molecular mechanics Poisson‐Boltzmann surface area (MMPBSA) method was used to calculate the binding free energy between α‐syn and 1‐ALPP using the gmx_MMPBSA package [27].

### 2.29 Intergenerational Assay

To investigate the intergenerational effects of E8 and 1‐ALPP, four A30P worms were initially cultured on NGM plates and subsequently transferred to individual plates seeded with either OP50 (control), E8, or 1‐ALPP. After five days of incubation, adult P0 worms (Day 1 of adulthood) were subjected to alkaline hypochlorite treatment (0.5% hypochlorous acid, 0.5 M NaOH) to remove the bacteria from the P0 generation and obtain eggs. These eggs were then cultured to generate the F1 offspring. The F1 generation was exclusively fed OP50 to eliminate direct exposure to E8 or 1‐ALPP, and the F2 generation was derived from F1 adults, ensuring no further contact with E8 beyond the P0 generation. The number of dendrites in the P0, F1, F2, and F3 generations was quantified to evaluate the persistence of intergenerational phenotypic changes.

### 2.30 In Vitro Fluorescent Labeling of Target Proteins With Fluorescein Isothiocyanate

Fluorescent labeling technology enables the visualization of target proteins through specific binding or covalent coupling of fluorescent dyes to target molecules. In this study, fluorescein isothiocyanate (FITC; Yuanye Bio‐Technology, Cat. S19127) was selected as the fluorescent dye to label target proteins via covalent conjugation with lysine residues. The labeling procedure was performed as follows: FITC (12.5 mg) was dissolved in 1 mL of DMSO (Solarbio, Cat. D8375) to prepare a 12.5 mg/mL (approximately 32 mM) stock solution. The target protein was mixed with an appropriate volume of the FITC stock solution in 1 mL of 0.2 M NaHCO_3_ buffer (pH ∼9.0), which provides the alkaline conditions necessary for efficient conjugation between FITC and protein amino groups. The reaction mixture was then brought to a final volume of 2 mL with double‐distilled water. The mixture was transferred to a 2 mL centrifuge tube, wrapped in aluminum foil to protect from light, and incubated at room temperature on an orbital shaker (∼200 rpm) for 2 h. Following incubation, unbound FITC was thoroughly removed by dialysis against an appropriate buffer using a membrane with a molecular weight cutoff of 8–14 kDa (Yuanye Bio‐Technology, Cat. MD1425). In the phase separation experimental system, the unlabeled protein stock solution was mixed with a small volume of fluorescently labeled protein solution at an approximate volume ratio of 10:1, achieving a labeled protein proportion of about 1% in the total protein content.

### 2.31 In Vitro Liquid–Liquid Phase Separation Assay

Fluorescence microscopy (Olympus BX53, Japan) was employed to investigate the liquid–liquid phase separation behavior of α‐syn under various experimental conditions. The phase separation reaction mixture consisted of 50 mM MES‐Tris buffer (pH 6.0), 250 mM NaCl, 10% PEG8000 (Solarbio, Cat. G0563), the target protein, and test drugs at designated concentrations. All samples were freshly prepared prior to observation. An aliquot (5–10 µL) of the sample was placed onto a clean coverslip, gently sealed with another coverslip to avoid bubble formation, and immediately transferred to the microscope stage for imaging. To minimize motion artifacts caused by micro‐flow beneath the coverslip and ensure high image fidelity, a rapid scanning mode was employed. Images were acquired using an excitation wavelength of 488 nm for detecting FITC‐labeled proteins, with a laser intensity of 50%, a scan speed of 4 µs/pixel, and a resolution of 1024 × 1024 pixels. This imaging setup enabled systematic evaluation of the effects of varying drug concentrations on the morphological characteristics, size distribution, and number density of α‐syn phase‐separated droplets.

### 2.32 Particle Size Measurement

The sample was filtered through a 0.22 µm membrane filter to remove large impurities and bubbles. Approximately 1 mL of the filtrate was collected and transferred to a disposable cuvette specifically for particle size measurement. Particle size was measured directly using a nanoparticle size analyzer (Zetasizer Pro, Malvern Panalytical Ltd., Malvern, UK). Each sample was measured 3–5 times, and the average value was used for subsequent data analysis.

### 2.33 Statistical Analysis

Statistical analysis was performed using GraphPad Prism 9.0 software. Comparisons between the two groups were evaluated for significant differences using a two‐tailed unpaired t‐test, while multiple group comparisons were detected using one‐way analysis of variance (ANOVA) followed by Tukey's post hoc test. All analyses were conducted based on the conventional significance threshold of α = 0.05 (*p* < 0.05 was considered significant). All data were presented with standardized graphic symbols to visually display the statistical significance results: ^*^ *p* < 0.05, ^**^ *p* < 0.01, ^***^ *p* < 0.001, ^****^ *p* < 0.0001, and ns indicated no statistical significance (*p* > 0.05).

## 3 Results

### 3.1 A Specific *E. coli* Strain E8 Alleviates PD‐Related Phenotypes in *C. elegans* Models

Transgenic overexpression of human α‐syn (A30P) in dopamine neurons of *C. elegans* (P*dat‐1*::α‐syn (A30P); P*cat‐1*::EYFP, hereafter referred to as the A30P PD model) leads to dendritic degeneration, recapitulating a key feature of PD (Figure 1A) [19]. Using this established A30P‐associated phenotype, we screened a collection of environmental and fermented vegetable‐derived bacterial isolates alongside the control strain *E. coli* OP50. Among these, one isolate, designated E8, markedly increased dendritic number in the A30P PD model (Figure 1B,C). 16S rRNA sequencing preliminarily indicated that strain E8 exhibited the highest sequence similarity to *E. coli* or *Shigella* species (Figure S1A). To verify E8 identity, we performed whole‐genome sequencing of the E8 strain. The Average Nucleotide Identity (ANI) values between E8 and the *E. coli* reference strains exceeded 96% (above the accepted species delineation threshold of 95%), unequivocally confirming that E8 belongs to the species *E. coli* (Figure S1B). Furthermore, phylogenetic analysis with multiple *E. coli* strains placed E8 in closer proximity to OP50 than to K‐12 or HT115 (Figure S1B). To evaluate whether this neuroprotective activity was strain‐specific, we tested several other *E. coli* strains (DH5α, Trans5α, TOP10, Trans10, and HT115). None reproduced the protective effect, indicating that the rescue of dendritic pathology is unique to this particular *E. coli* subtype (Figure 1D). To exclude the possibility that E8 acts by downregulating the P*dat‐1* promoter, which drove A30P expression in dopaminergic neurons, we measured P*dat‐1*::GFP fluorescence intensity. Interestingly, E8 treatment led to a modest increase, rather than a decrease, in P*dat‐1* expression (Figure 1E), suggesting that its neuroprotective function operates independently of transgene suppression. Furthermore, E8 administration did not alter developmental timing from egg to larval stage 4 (L4) (Figure 1F), implying that it does not influence the growth rate.

**FIGURE 1:**
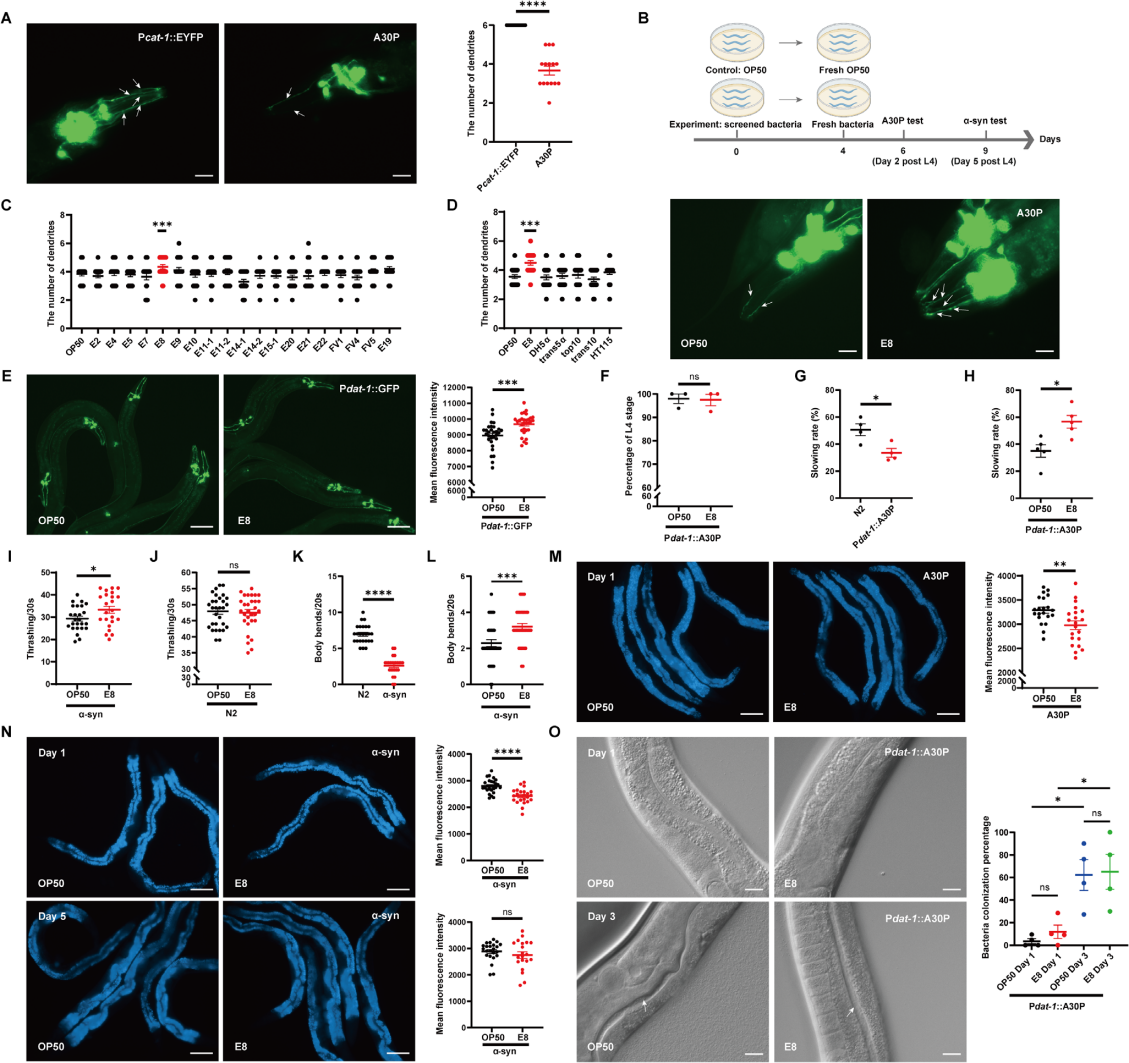
E8 alleviates PD‐related phenotypes in *C. elegans* models. (A) Representative fluorescence images and quantitative analysis of the number of dendrites in the PD model and the control P*cat‐1*::EYFP. Arrows indicate dendrites of head neurons. Scale bar = 20 µm, Two‐tailed unpaired Student's t‐test, *n* = 15–20. (B) Schematic illustrations for the feeding bacterial treatment. (C) Unbiased screening of bacteria that improved the degeneration of dendrites in the PD model. One‐way ANOVA followed by Tukey's post hoc test, *n* = 14–20. (D) Statistical graph and representative fluorescence images demonstrating *E. coli*‐mediated improvement of dendrite degeneration in the PD model. Arrows indicate the dendrites of head neurons. Scale bar = 20 µm. One‐way ANOVA followed by Tukey's post hoc test, *n* = 18–20. (E) Representative fluorescence images and quantitative analysis of the fluorescence intensity of P*dat‐1*::GFP treated with OP50 or E8. Scale bar = 100 µm. Two‐tailed unpaired Student's t‐test, *n* = 30. (F) Growth rate of PD models treated with OP50 or E8. Two‐tailed unpaired Student's t‐test. *n* = 3 (biological replicates). (G) Statistical analysis of the slowing rate of N2 and PD models. Two‐tailed unpaired Student's t‐test, *n* = 4 (biological replicates). (H) Statistical analysis of the slowing rate of PD models for OP50 and E8. Two‐tailed unpaired Student's t‐test. *n* = 5 (biological replicates). (I) Statistical analysis of α‐syn PD model thrashing after OP50 or E8 treatment. Two‐tailed unpaired Student's t‐test. *n* = 23–25. (J) Statistical analysis of N2 thrashing after OP50 or E8 treatment. Two‐tailed unpaired Student's t‐test, *n* = 30. (K) Statistical analysis of body bends of N2 and α‐syn PD model. Two‐tailed unpaired Student's t‐test, *n* = 25. (L) Statistical analysis of α‐syn PD model body bends after OP50 or E8 treatment. Two‐tailed unpaired Student's t‐test, *n* = 35. (M) Fluorescence and statistical analysis of Day 1 A30P PD model lipofuscin levels following OP50 or E8 treatment. Scale bar = 100 µm, Two‐tailed unpaired Student's t‐test, *n* = 21. (N) Fluorescence and statistical analysis of Day 1/5 α‐syn PD model lipofuscin levels after OP50 or E8 treatment. Scale bar = 100 µm, Two‐tailed unpaired Student's t‐test, *n* = 20–27. (O) Colonization percentage of OP50 or E8 in the P*dat‐1*::A30P PD model intestinal tract at Day 1 (D1) or Day 3 (D3). Arrows indicate the colonized bacteria in the gut. Scale bar = 20 µm. One‐way ANOVA followed by Tukey's post hoc test, *n* = 4 (biological replicates). Data were presented as mean ± SEM. ns, not significant (*p* > 0.05); ^*^ *p* < 0.05; ^**^ *p* < 0.01; ^***^ *p* < 0.001; ^****^ *p* < 0.0001.

To test the efficacy of E8 in a second PD model, we established a 6‐OHDA acute neurotoxicity assay as described [28]. L3‐stage BY250 (P*dat‐1*::GFP) animals were treated with 50 mM 6‐OHDA for 1 h, which induced dendritic beading without complete loss. At 72 h post‐treatment, 6‐OHDA caused extensive dendritic breaks in CEP neurons, whereas E8 or L‐DOPA (positive control) significantly reduced this damage (Figure S2A). Thus, E8 is neuroprotective in both transgenic and chemically induced PD models.

In *C. elegans*, locomotion is achieved through sinusoidal bending of the body, and the bending frequency determines movement speed. Upon encountering food (e.g., bacteria), worms reduce this frequency to slow down and feed more efficiently, a behavior known to be mediated by dopaminergic neurotransmission. Consistent with this, the P*dat‐1*::A30P PD model, which is characterized by dopaminergic neurodegeneration, displays a specific defect in this food‐sensing response, as evidenced by a reduced slowing rate (Figure 1G) [19]. E8 administration rescued the slowing rate defect in the P*dat‐1*::A30P PD model (Figure 1H), suggesting that it may ameliorate the underlying dopaminergic dysfunction.

To characterize the novel *E. coli* E8 isolate and its potential role in mitigating PD pathology, we systematically compared its properties with the control strain *E. coli* OP50. E8 exhibited similar colony‐forming capacity (11.33 × 10^8^ CFU) compared to OP50 (11.43 × 10^8^ CFU) (Figure S1C) but was preferentially selected by the P*dat‐1*::A30P model or N2 in a food choice assay (Figure S1D,E). Importantly, E8 treatment resulted in comparable progeny production in the PD model (234.3 vs. 261.5 offspring per animal on E8 and OP50, respectively), indicating no effect on fertility (Figure S1F). The neuroprotective effect of E8 was recapitulated by its lysate obtained via ultrasonication (Figure S1G), indicating cell‐associated activity, while its culture supernatant alone rescued dendritic pathology in the A30P model (Figure S1H), suggesting secretion of active metabolites. Heat sensitivity assays revealed that pasteurization (70°C, 30 min) preserved E8's neuroprotective capacity (Figure S1I), whereas autoclaving (121°C, 20 min) abolished it (Figure S1J).

To comprehensively assess the impact of E8 beyond its neuroprotective effects, we evaluated multiple physiological parameters in *C. elegans*. Compared with the OP50 control, E8 treatment did not affect the thrashing rate of wild‐type N2 worms in M9 buffer (Figure 1J). In contrast, it significantly increased the thrashing rate of the PD model strain NL5901, which expresses α‐syn::YFP in body wall muscle (hereafter referred to as the α‐syn PD model) (Figure 1I). Furthermore, the α‐syn PD worms exhibited a marked reduction in body bends relative to N2 controls (Figure 1K), confirming the reproducibility of the locomotor defect and ruling out artifacts associated with the liquid‐based assay. Importantly, E8 treatment significantly ameliorated this defect (Figure 1L), in line with the thrashing result and suggesting improved motor function and health span in this PD model. Under heat stress, E8 did not significantly affect the survival rate of either the PD model or wild‐type N2 worms, though a modest trend was observed (Figure S1K,L). Despite these benefits, E8 did not extend the lifespan of either the PD model or wild‐type N2 worms (Figure S1M,N). Additionally, we assessed lipofuscin accumulation, which is often used as a marker of health or the rate of aging [29]. E8 reduced lipofuscin levels in two PD models, A30P and α‐syn, at Day 1 post‐L4 (Figure 1M,N), but this effect was not sustained, as no significant reduction was observed in α‐syn worms at Day 5 post‐L4 (Figure 1N). Finally, we investigated the gut colonization capacity of E8, a key feature for probiotic functionality. At Day 1 post‐L4, both OP50 and E8 were present at low levels in the worm gut, but colonization increased substantially by Day 3 post‐L4, with no significant difference between the two strains (Figure 1O). This suggests that E8 colonizes the *C. elegans* intestine comparably to OP50.

### 3.2 E8 Ameliorates the Phenotype of PD Model in *C. elegans* Through Enhancing Autophagy

We next asked whether E8 could mitigate α‐syn aggregation, a key feature of PD pathogenesis, using the NL5901 strain expressing α‐syn::YFP in body wall muscle. E8 significantly reduced α‐syn::YFP aggregate number (Figure 2A), indicating that its protection of dopaminergic dendrites likely results from the inhibition of α‐syn aggregation. Then, we employed a transgenic strain, P*dat‐1*::α‐syn(A30P)::GFP; P*dat‐1*::mCherry, which enables direct in vivo visualization of α‐syn in dopaminergic neurons. Using this strain, we observed prominent punctate signals in the dendritic processes. Notably, these puncta were absent in the P*dat‐1*::GFP control animals, suggesting that they represent α‐syn aggregates. Importantly, treatment with E8 significantly reduced the number of these aggregates in dopaminergic dendrites (Figure S2B), suggesting that E8 suppresses α‐syn accumulation in vivo.

**FIGURE 2:**
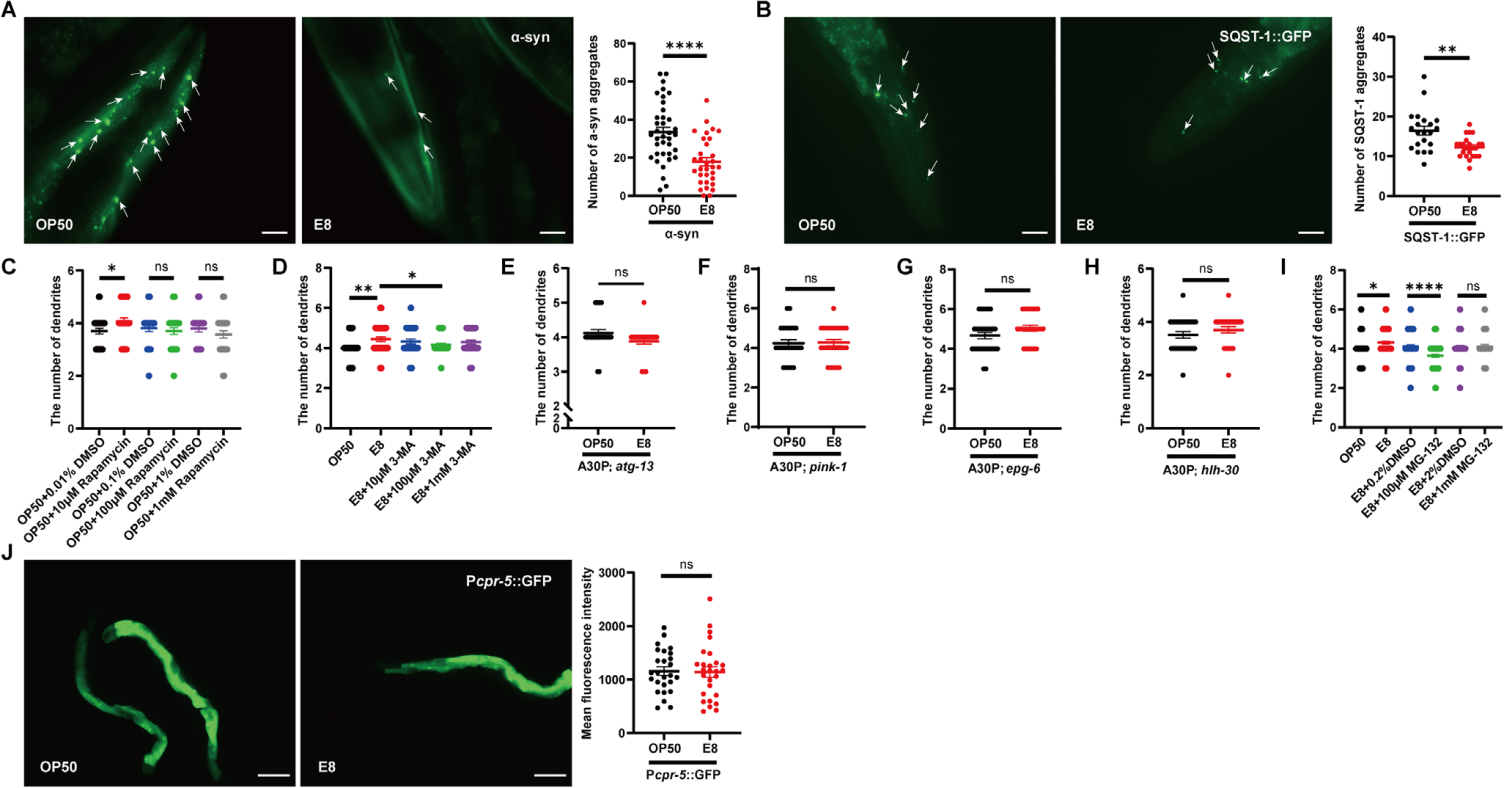
E8 ameliorates the neurodegeneration of the PD model via autophagy. (A) Representative fluorescence images and quantitative analysis of α‐syn protein aggregation after OP50 or E8 treatment. Arrows indicate α‐syn protein aggregation. Scale bar = 20 µm, Two‐tailed unpaired Student's t‐test, *n* = 32–37. (B) Representative fluorescence images and quantification of SQST‐1::GFP puncta following treatment with OP50 or E8. Arrows indicate the autophagy substrate SQST‐1::GFP. Scale bar = 20 µm. Two‐tailed unpaired Student's t‐test, *n* = 21–23. (C) Quantification of dendritic numbers treated with various rapamycin concentrations. One‐way ANOVA followed by Tukey's post hoc test, *n* = 20–30. (D) Quantification of dendritic numbers treated with various 3‐MA concentrations. One‐way ANOVA followed by Tukey's post hoc test, *n* = 33–38. (E–H) Quantification of the dendrite numbers in A30P; *atg‐13*, A30P; *pink‐1*, A30P; *epg‐6*, and A30P; *hlh‐30* after treatment with OP50 or E8. Two‐tailed unpaired Student's t‐test, *n* = 24–30. (I) Quantification of dendritic numbers treated with various MG‐132 concentrations. One‐way ANOVA followed by Tukey's post hoc test, *n* = 29–100. (J) Representative fluorescence images and fluorescence intensity quantitation of P*cpr‐5*::GFP after treatment with OP50 or E8. Scale bar = 100 µm. Two‐tailed unpaired Student's t‐test, *n* = 26–27. Data were presented as mean ± SEM. ns, not significant (*p > *0.05); ^*^ *p* < 0.05; ^**^ *p* < 0.01; ^****^ *p* < 0.0001.

Given the critical role of autophagy in the degradation of abnormal protein aggregates, we hypothesized that E8 might mitigate α‐syn accumulation by enhancing autophagic activity. To test this hypothesis, we quantified SQST‐1::GFP puncta, an established autophagic substrate and reliable marker of autophagy, and found a significant reduction in puncta number following E8 treatment (Figure 2B). This observation directly supports the notion that E8 reduces α‐syn aggregation through the activation of autophagy. To further validate the essential role of autophagy in E8‐mediated neuroprotection, we performed complementary pharmacological and genetic experiments. First, we treated the PD model with rapamycin, a well‐characterized autophagy agonist, to assess whether direct autophagy activation could recapitulate E8's protective effects on the PD phenotype. As anticipated, treatment with 10 µM rapamycin significantly increased dendrite numbers in the A30P PD model (Figure 2C), mirroring the neuroprotective effect of E8. Conversely, co‐treatment with 3‐methyladenine (3‐MA), a specific autophagy inhibitor, abrogated the beneficial effects of E8 (Figure 2D), confirming that E8's function is dependent on autophagy. To further corroborate these findings, we examined the effect of E8 in PD models harboring loss‐of‐function mutations in autophagy‐related genes, including *atg‐13*, *pink‐1*, *epg‐6*, and *hlh‐30*. Notably, mutations in all these autophagy genes blocked E8's ability to ameliorate dendrite defects (Figure 2E–H). Collectively, these pharmacological and genetic lines of evidence suggest that E8 exerts its neuroprotective effects in the PD model by enhancing autophagy.

Given the proteasome's pivotal role in maintaining cellular protein homeostasis, we hypothesized that E8 acts by augmenting proteasomal activity to facilitate clearance of pathological α‐syn aggregates. To mechanistically validate this, we employed a pharmacological inhibition strategy by co‐administering the selective proteasome inhibitor MG‐132 with E8. This intervention resulted in a significant attenuation of E8's neuroprotective effects against α‐syn‐induced toxicity (Figure 2I). It provides compelling evidence that E8 exerts its beneficial effects through the proteasome‐mediated protein degradation pathway.

As the primary cellular degradation center responsible for substrate recycling [30], we monitored the lysosomal surveillance response (LySR) pathway—characterized by enhanced lysosomal activity and protein aggregate clearance [31] using the LySR reporter strain P*cpr‐5*::GFP. Notably, E8 treatment did not alter P*cpr‐5*::GFP fluorescence intensity compared to OP50 controls (Figure 2J), demonstrating that E8's neuroprotective effects occur independently of LySR activation.

### 3.3 Joint Analysis of Untargeted Transcriptomics and Metabolomics of E8‐Treated PD Model

To further elucidate the underlying mechanism of E8 in the PD model, we conducted an integrated analysis of untargeted transcriptomics and metabolomics. The experimental design included three distinct groups: (1) the Cat‐OP group (control worms fed with OP50 bacteria), (2) the A30P‐OP group (PD model worms fed with OP50 bacteria), and (3) the A30P‐E8 group (PD model worms treated with E8). Initially, transcriptome and metabolome principal component analysis (PCA) revealed distinct separation trends among the sample groups (Figure 3A,B). Subsequently, a cluster heatmap (Figure 3C,D) and a Sankey diagram (Figure 3E,F) were generated for the top 30 biomarkers, highlighting differences in expression patterns of characteristic molecular groups and the relationships between key differentially expressed genes and metabolites. A Venn diagram illustrated the intersection of common pathways targeted by the two groups, showing 9 overlapping pathways between the control and model groups and 40 between the control and E8‐fed PD model groups (Figure 3G,H). An enrichment bubble plot further demonstrated that these pathways predominantly involve processes such as axon regeneration, autophagy, lysosomes, and the mTOR signaling pathway (Figure 3I,J), suggesting that E8 may regulate these pathways to coordinate metabolic homeostasis and physiological adaptation in the host.

**FIGURE 3:**
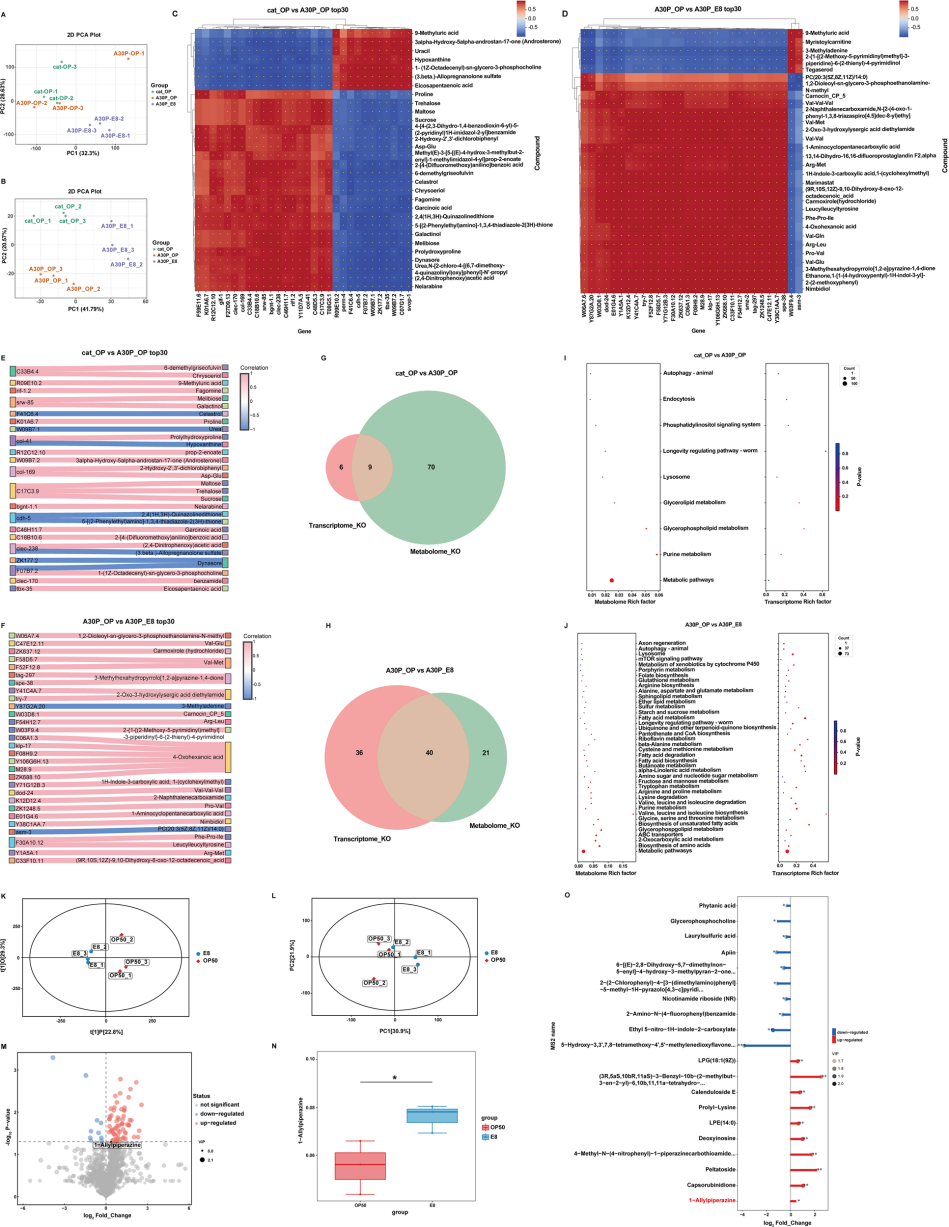
Joint analysis of non‐targeted transcriptome and metabolome of E8‐treated worms. (A) Transcriptome Principal Component Analysis (PCA) score plots for Cat‐OP (control worm treated with control bacteria OP50), A30P‐OP, and A30P‐E8. (B) Metabolome Principal Component Analysis (PCA) score plots for Cat‐OP (control worm treated with control bacteria OP50), A30P‐OP, and A30P‐E8. (C) The correlation clustering heatmap of the top 30 differential metabolites and differential genes for Cat‐OP and A30P‐OP. (D) The correlation clustering heatmap of the top 30 differential metabolites and differential genes for A30P‐OP and A30P‐E8. (E) The correlation network Sankey diagram of the top 30 differential metabolites and differential genes between Cat‐OP and A30P‐OP. (F) The correlation network Sankey of the top 30 differential metabolites and differential genes between A30P‐OP and A30P‐E8. (G) The KEGG enrichment Venn diagram of the differential metabolites and differential genes between Cat‐OP and A30P‐OP. (H) The KEGG enrichment Venn diagram of the differential metabolites and differential genes between A30P‐OP and A30P‐E8. (I) The KEGG enrichment bubble plots of the differential metabolites and differential genes between Cat‐OP and A30P‐OP. (J) The KEGG enrichment bubble plots of the differential metabolites and differential genes between A30P‐OP and A30P‐E8. (K) Orthogonal partial least squares discriminant analysis (OPLS‐DA) plot. (L) Score plot of principal component analysis (PCA). (M) Volcano plot comparing the OP50 and E8 groups, with red representing upregulated metabolites and blue representing downregulated metabolites. (N) Plot of the relative content of 1‐allylpiperazine in OP50 and E8 bacteria. (O) Bar chart displaying the fold changes of the selected metabolites. ^*^ *p* < 0.05.

To systematically characterize the metabolic differences between OP50 and E8, we conducted an untargeted metabolomics analysis comparing E8 with OP50. The orthogonal partial least squares discriminant analysis (OPLS‐DA) revealed a clear separation between the OP50 and E8 groups (Figure 3K), which was further corroborated by principal component analysis (PCA) demonstrating significant intergroup separation (Figure 3L), indicating substantial differences in their overall molecular profiles. The volcano plot analysis identified 68 significantly upregulated metabolites and 10 downregulated metabolites in E8 compared to OP50 (Figure 3M), highlighting the distinct metabolic signatures of the E8 strain.

### 3.4 E8 Metabolite 1‐ALPP Ameliorates the Neurodegeneration of PD Model by Enhancing Autophagy

To identify the functional metabolites of E8, we screened for metabolites exhibiting elevated abundance in E8 relative to the control bacterium OP50. Among more than 30 candidates evaluated (Figure 4A,B, not all candidates are listed), one metabolite, 1‐ALPP, was identified. At a concentration of 1%, 1‐ALPP increased dendrite numbers in the PD model (Figure 4C) and suppressed α‐syn aggregation (Figure 4G). Furthermore, 1% 1‐ALPP rescued the slowing rate defect in the PD model (Figure 4D), suggesting a potential restoration of dopaminergic deficits. In addition, treatment with 1% 1‐ALPP elevated the thrashing rate but not body bends of α‐syn PD model compared to OP50 control (Figure 4E,F), suggesting an improvement in health span in the PD model. Metabolomic analysis confirmed that 1‐ALPP levels were higher in the E8 group, with a 1.37‐fold increase relative to OP50 (Figure 3N,O).

**FIGURE 4:**
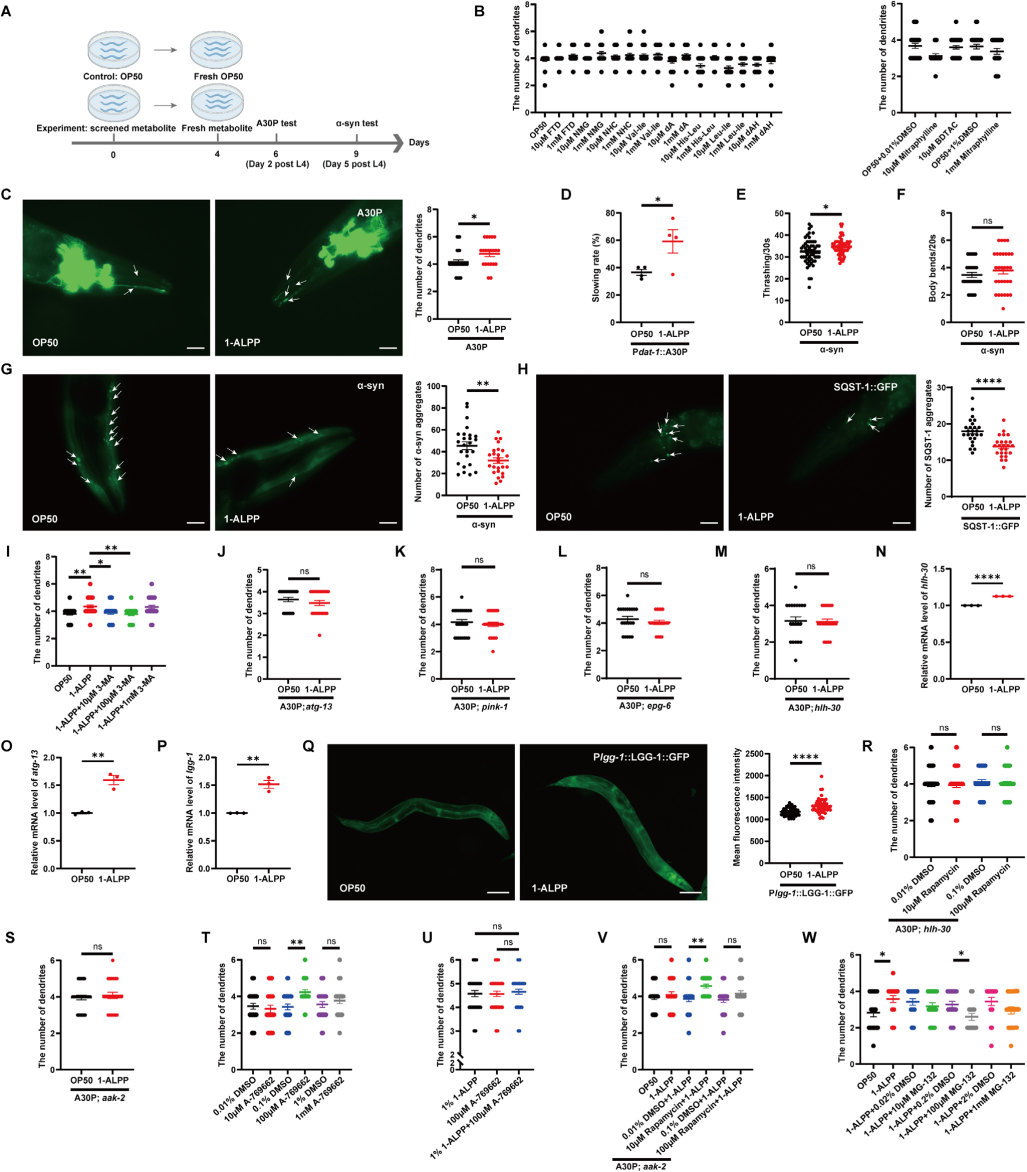
1‐ALPP ameliorates the phenotype of the PD model by enhancing autophagy. (A) Schematic illustrations for the metabolite treatment. (B) Quantification of dendrite numbers of A30P PD model following treatment with E8 small molecule metabolites. One‐way ANOVA followed by Tukey's post hoc test, *n* = 15–32. (C) Representative fluorescence images and quantification of dendrite numbers in the PD model after feeding with OP50 or 1‐ALPP. Arrows indicate dendrites of head neurons. Scale bar = 20 µm, Two‐tailed unpaired Student's t‐test, *n* = 21–27. (D) Statistical analysis of the slowing rate of the PD model for OP50 and 1‐ALPP. Two‐tailed unpaired Student's t‐test, *n* = 4 (biological replicates). (E) Statistical analysis of the effect of OP50 or 1‐ALPP on PD model thrashing. Two‐tailed unpaired Student's t‐test, *n* = 54. (F) Statistical analysis of PD model body bends after OP50 or 1‐ALPP treatment. Two‐tailed unpaired Student's t‐test, *n* = 30. (G) Representative fluorescence images and quantification of α‐syn aggregation numbers following treatment with OP50 or 1‐ALPP. Arrows indicate α‐syn protein aggregates. Scale bar = 20 µm, Two‐tailed unpaired Student's t‐test, *n* = 25. (H) Representative fluorescence images and quantification of autophagy substrate SQST‐1::GFP numbers after treatment with OP50 or 1‐ALPP. Arrows indicate the autophagy substrate. Scale bar = 20 µm, Two‐tailed unpaired Student's t‐test, *n* = 25. (I) Quantification of dendritic numbers across different 3‐MA concentrations and 1% 1‐ALPP. One‐way ANOVA followed by Tukey's post hoc test, *n* = 31–34. (J–M) Quantification of the dendrite numbers in A30P; *atg‐13*, A30P; *pink‐1*, A30P; *epg‐6*, A30P; *hlh‐30* after treatment with OP50 or 1‐ALPP. Two‐tailed unpaired Student's t‐test. *n* = 20–25. (N–P) qPCR showing the relative mRNA expression levels of *hlh‐30*, *atg‐13*, and *lgg‐1*. Two‐tailed unpaired Student's t‐test, *n* = 3 (biological replicate). (Q) Representative fluorescence images and quantification of P*lgg‐1*::LGG‐1::GFP after treatment with OP50 or 1‐ALPP. Scale bar = 100 µm. Two‐tailed unpaired Student's t‐test. *n* = 54. (R) Quantification of dendritic numbers of A30P; *hlh‐30* across different rapamycin concentrations. One‐way ANOVA followed by Tukey's post hoc test, *n* = 27–50. (S) Quantification of the dendrite numbers in A30P; *aak‐2* after treatment with OP50 or 1‐ALPP. Two‐tailed unpaired Student's t‐test, *n* = 24–28. (T) Quantification of dendritic numbers across different A‐769662 concentrations. One‐way ANOVA followed by Tukey's post hoc test, *n* = 25–30. (U) Quantification of dendritic numbers across 1‐ALPP, A‐769662, and 1‐ALPP+A‐769662. One‐way ANOVA followed by Tukey's post hoc test, *n* = 40–53. (V) Quantification of dendritic numbers of A30P; *aak‐2* across rapamycin+1‐ALPP. One‐way ANOVA followed by Tukey's post hoc test, *n* = 24–30. (W) Quantification of dendritic numbers across different concentrations of MG‐132+1‐ALPP. One‐way ANOVA followed by Tukey's post hoc test, *n* = 15–20. Data were presented as mean ± SEM. ns, not significant (*p* > 0.05); ^*^ *p* < 0.05; ^**^ *p* < 0.01; ^****^ *p* < 0.0001.

Building on the above findings that E8 acts via autophagy, we next investigated whether 1‐ALPP employs a similar autophagy‐dependent mechanism to exert its neuroprotective effects. First, we assessed autophagic activity following 1‐ALPP treatment and found that 1‐ALPP significantly promoted autophagy, as evidenced by a reduction in SQST‐1::GFP puncta (Figure 4H), consistent with the autophagy‐enhancing effect of E8. To confirm the dependence of 1‐ALPP's function on autophagy, we used the autophagy inhibitor 3‐MA: co‐treatment with 3‐MA effectively counteracted the neuroprotective effects of 1‐ALPP (Figure 4I), mirroring the results observed with E8. Further genetic validation showed that the protective effect of 1‐ALPP against neurodegeneration in the PD model was abolished in PD models carrying loss‐of‐function mutations in the autophagy‐related genes *atg‐13*, *pink‐1*, *epg‐6*, and *hlh‐30* (Figure 4J–M). Collectively, these data strongly suggest that 1‐ALPP, like E8, ameliorates neurodegeneration in the PD model by enhancing autophagy.

To dissect the molecular mechanism by which 1‐ALPP activates autophagy, we focused on the autophagy‐associated transcription factor HLH‐30 (the *C. elegans* homolog of TFEB). This focus was based on our observation that the *hlh‐30* mutation abrogated 1‐ALPP's ability to ameliorate neurodegeneration in the PD model (Figure 4M), suggesting that 1‐ALPP acts through HLH‐30‐mediated autophagy. Furthermore, qPCR analysis revealed that the expression levels of *hlh‐30*, its downstream targets *atg‐13* (whose mutant is known to block 1‐ALPP function), and *lgg‐1* were all significantly elevated (Figure 4N–P). Consistent with this, monitoring of P*lgg‐1*::LGG‐1::GFP transgenic worms showed a corresponding increase in *lgg‐1* level (Figure 4Q), corroborating the qPCR findings. Next, we sought to identify the upstream regulator of HLH‐30 in the 1‐ALPP‐mediated pathway. Given that the mammalian target of rapamycin (mTOR) is a well‐documented negative regulator of HLH‐30/TFEB [32], we hypothesized that 1‐ALPP might exert its effects by inhibiting mTOR signaling. A key prediction of this hypothesis is that another mTOR inhibitor would recapitulate the neuroprotective effects of 1‐ALPP in the PD model. This prediction was validated by our finding that the mTOR inhibitor rapamycin effectively ameliorated the PD phenotype (Figure 2C). Notably, the *hlh‐30* mutation blocked rapamycin's neuroprotective effect (Figure 4R), confirming that HLH‐30 functions downstream of mTOR in mediating PD phenotype amelioration.

We then further explored how 1‐ALPP inhibits mTOR signaling. Previous studies have shown that AMP‐activated protein kinase (AMPK) can directly inhibit mTOR function [32]; thus, we hypothesized that 1‐ALPP activates AMPK to inhibit mTOR, thereby enhancing autophagy and ameliorating the PD phenotype. To test this, we examined the effect of 1‐ALPP in PD models carrying a loss‐of‐function mutation in *aak‐2*, a gene encoding a catalytic subunit of AMPK in *C. elegans*. As expected, *aak‐2* mutation blocked the neuroprotective effect of 1‐ALPP (Figure 4S), supporting our hypothesis. Consistent with this, treatment with the AMPK activator A‐769662 mimicked the neuroprotective effect of 1‐ALPP in the PD model (Figure 4T). Furthermore, co‐treatment with A‐769662 and 1‐ALPP did not further enhance the protective effect (Figure 4U), suggesting that 1‐ALPP and A‐769662 act in the same signaling pathway.

To further validate the AMPK‐mTOR‐HLH‐30 autophagy pathway of 1‐ALPP in PD phenotype amelioration, we supplemented rapamycin to 1‐ALPP‐treated α‐syn A30P; *aak‐2* mutant worms. The results showed that rapamycin treatment reversed the loss of 1‐ALPP's neuroprotective effect caused by the *aak‐2* mutation (Figure 4V), confirming that mTOR functions downstream of AMPK in this pathway. Taken together, all these findings collectively support the conclusion that 1‐ALPP ameliorates the PD phenotype by activating AMPK, which in turn inhibits mTOR signaling, promotes HLH‐30‐mediated autophagy, and ultimately mitigates neurodegeneration.

Co‐administering the selective proteasome inhibitor MG‐132 with 1‐ALPP resulted in a significant attenuation of its neuroprotective effects against α‐syn‐induced toxicity (Figure 4W), suggesting 1‐ALPP exerts its beneficial effects through the proteasome‐mediated protein degradation pathway.

### 3.5 E8 Metabolite 1‐ALPP Inhibits α‐syn Fibrillogenesis and Alleviates its Cytotoxicity

ThT molecules specifically bind to β‐sheet structures of α‐syn aggregates and exhibit enhanced fluorescence intensity. The content of β‐sheet structure of α‐syn aggregates is significantly increased during the fibrillation process. Thus, this property allows the aggregation level of α‐syn to be quantitatively characterized by monitoring ThT fluorescence intensity [33]. Herein, the inhibitory effect of 1‐ALPP against α‐syn fibrillation was also explored using ThT fluorescence. For clear comparison, all fluorescence intensity data were normalized, with the maximum fluorescence intensity achieved by α‐syn incubated alone set at 100%. When incubated with 50 µM α‐syn alone at 37°C for 10 d, the ThT fluorescence profile showed a lag phase during the previous 4 d. Then, a fast growth phase occurred between 4 and 6 d, and a steady equilibrium phase after 9 d (Figure 5A), consistent with previous studies [22, 34]. To evaluate 1‐ALPP inhibitory effect against α‐syn fibrillogenesis, different concentrations of 1‐ALPP were co‐incubated with 50 µM α‐syn. At a 1:0.5 molar ratio of α‐syn to 1‐ALPP, a decline in fluorescence intensity of approximately 51.19% was observed in comparison to the pure α‐syn. Increasing the concentration of 1‐ALPP to a 1:1 molar ratio resulted in a further reduction in fluorescence intensity. When the ratio reached 1:2, a significant decrease in fluorescence intensity to 16.45% was observed (Figure 5B). The results indicate that 1‐ALPP effectively inhibits α‐syn fibrillation, with its inhibitory effect increasing with concentration.

**FIGURE 5:**
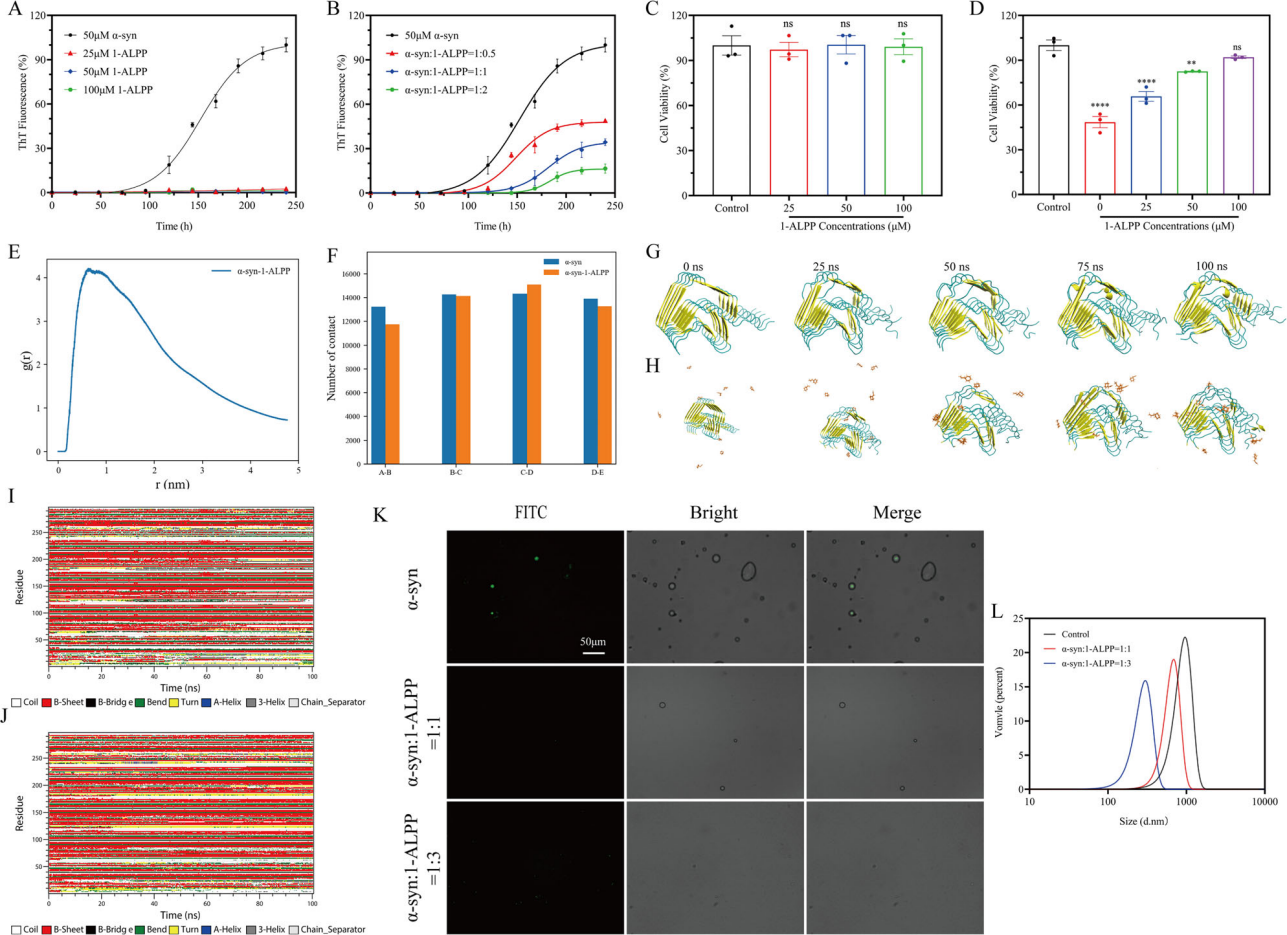
1‐ALPP inhibits α‐syn fibrillogenesis using in vitro and in silico studies. (A) Relative ThT fluorescence intensity as a function of incubation time at different concentrations of 1‐ALPP during α‐syn incubation. One‐way ANOVA followed by Tukey's post hoc test, *n* = 3. (B) ThT fluorescence intensity in the presence of 1‐ALPP at different concentrations. One‐way ANOVA followed by Tukey's post hoc test, *n* = 3. (C) Cytotoxicity of 1‐ALPP measured by CCK‐8 assay. One‐way ANOVA followed by Tukey's post hoc test, *n* = 3. (D) The alleviation of α‐syn‐induced cytotoxicity by 1‐ALPP assessed by the CCK‐8 assay. One‐way ANOVA followed by Tukey's post hoc test, *n* = 3. (E) Radial distribution function (RDF) between 1‐ALPP and α‐syn. (F) Inter‐chain contact numbers of the α‐syn pentamer in the presence and absence of 1‐ALPP. (G) Typical snapshots of the α‐syn pentamer during the 100 ns MD simulation in the pure α‐syn system. (H) The typical snapshots were obtained during the 100 ns MD simulation in the 1‐ALPP/α‐syn complex. Secondary structure evolution of the α‐syn pentamer in the presence (I) and absence (J) of 1‐ALPP. (K) Effect of 1‐ALPP on LLPS. FITC‐labeled α‐syn (green) at a concentration of 30 µM was incubated in buffer solution in the absence (control) or presence of 1‐ALPP. Samples were prepared and immediately observed under a fluorescence microscope with an excitation wavelength of 488 nm. Scale bar: 50 µm. (L) Particle size changes in the LLPS system. Data were presented as mean ± SEM. Statistical significance was determined by one‐way ANOVA followed by Tukey's post hoc test. Significance levels are indicated as: ns, not significant (*p* > 0.05); ^**^ *p* < 0.01; ^****^ *p* < 0.0001.

The neurotoxicity of α‐syn aggregates was evaluated in vitro using rat pheochromocytoma (PC12) cells, and cell viability was assessed via the CCK‐8 assay. To preclude any potential interference, we first examined the intrinsic cytotoxicity of 1‐ALPP on PC12 cells. It indicated that 1‐ALPP showed almost no cytotoxicity within the concentration range studied (Figure 5C). Figure 5D showed the inhibitory capacity of 1‐ALPP against α‐syn‐induced cytotoxicity in PC12 cells using the CCK‐8 assay. In the absence of 1‐ALPP, after 11 d of incubation of 50 µM α‐syn with PC12 cells, α‐syn caused a significant reduction in cellular viability of 49.50% relative to the untreated group (Figure 5D). In the presence of different concentrations of 1‐ALPP, they all showed protective effects from α‐syn induced cytotoxicity to some extent, and the protective effects were concentration‐dependent. As compared to an amyloid‐induced cell viability of 49.50%, 1‐ALPP cell viability rates were improved by 16.28% at 25 µM, 32.97% at 50 µM, 42.49% at 100 µM. Therefore, these results demonstrate that 1‐ALPP is an effective inhibitor that can reduce α‐syn‐induced cytotoxicity.

### 3.6 Inhibitory Mechanism of E8 Metabolite 1‐ALPP Probed by Molecular Dynamics Simulation

During the simulation, 1‐ALPP was predominantly located around 1 nm from α‐syn (Figure 5E). In the presence of 1‐ALPP, the number of inter‐chain contacts of the α‐syn pentamer was markedly reduced, indicating that 1‐ALPP loosened the structure of α‐syn and hindered its aggregation (Figure 5F). To evaluate the binding affinity between 1‐ALPP and α‐syn, MM/PBSA calculations were performed (Table 1). The binding energy was −53.67 kcal/mol, suggesting that 1‐ALPP forms a stable complex with α‐syn. Figure 5G,H showed the trajectories of α‐syn alone and the 1‐ALPP/α‐syn complex. The α‐syn pentamer maintained its overall structure during the simulation, with β‐sheets remaining the dominant secondary structure (Figure 5G). In contrast, in the 1‐ALPP/α‐syn system, 1‐ALPP gradually approached α‐syn during the first 50 ns and bound stably during the last 50 ns, resulting in a looser structure and a reduced β‐sheet content (Figure 5H). The RMSD, SASA, and RMSF results (Table 2) further support that 1‐ALPP disrupted the structural stability of α‐syn. Figure 5I,J showed the secondary structure evolution of the 1‐ALPP/α‐syn and α‐syn systems, respectively. Under the influence of 1‐ALPP, extensive β‐sheet regions, particularly residues 10–40, 60–70, 120–140, 200, and 260–270, were converted into random coils, thereby destabilizing α‐syn and ultimately reducing its aggregation propensity.

**TABLE 1:** Binding energy during the last 10 ns of the simulation.

Energy components	1‐ALPP‐α‐syn complex (kcal/mol)
ΔG_complex_	1152.10 ± 48.62
ΔG_receptor_	709.11 ± 45.77
ΔG_ligand_	496.66 ± 9.64
ΔG_bind_ ^a^	−53.67 ± 11.40

^a^ ΔG_bind_ = ΔG_complex_ − (ΔG_receptor_ + ΔG_ligand_).

**TABLE 2:** Root Mean Square Deviation (RMSD), Solvent Accessible Surface Area (SASA), and Root Mean Square Fluctuation (RMSF) of the α‐syn system and the 1‐ALPP–α‐syn system during the simulation.

	RMSD		SASA		RMSF	
	α‐syn	α‐syn‐1‐ALPP	α‐syn	α‐syn‐1‐ALPP	α‐syn	α‐syn‐1‐ALPP
whole	0.42 ± 0.05	0.50 ± 0.11	136.68 ± 3.66	140.74 ± 3.14	0.14 ± 0.11	0.17 ± 0.18
A	0.62 ± 0.10	0.76 ± 0.26	60.55 ± 2.00	63.50 ± 3.45	0.21 ± 0.13	0.34 ± 0.24
B	0.38 ± 0.06	0.37 ± 0.09	62.06 ± 0.95	65.00 ± 1.43	0.12 ± 0.08	0.16 ± 0.12
C	0.23 ± 0.03	0.27 ± 0.04	60.96 ± 1.15	63.41 ± 1.48	0.10 ± 0.07	0.12 ± 0.08
D	0.24 ± 0.02	0.32 ± 0.04	62.25 ± 1.15	62.98 ± 1.07	0.12 ± 0.07	0.12 ± 0.08
E	0.37 ± 0.05	0.46 ± 0.06	62.11 ± 2.04	61.94 ± 1.34	0.17 ± 0.10	0.14 ± 0.07

In conclusion, our MD simulations reveal that 1‐ALPP forms a stable complex with α‐syn, with a binding energy of −53.67 kcal/mol. During the simulation, 1‐ALPP progressively approaches and binds to α‐syn, inducing transitions from β‐sheet to random coil in several key regions and significantly disrupting the protein's secondary structure. Furthermore, 1‐ALPP decreases the structural stability and compactness of α‐syn by reducing inter‐chain contacts, making the overall assembly more flexible and effectively inhibiting α‐syn aggregation.

### 3.7 E8 Metabolite 1‐ALPP Suppresses α‐syn Liquid–Liquid Phase Separation

Liquid‐liquid phase separation (LLPS) is the initial step in the pathological aggregation of α‐syn. This process is driven by weak multivalent interactions, finely regulated by microenvironmental factors, and is closely associated with protein misfolding and amyloid fibril formation. To investigate the effect of 1‐ALPP on α‐syn LLPS, FITC‐labeled α‐syn (30 µM) was used to observe phase separation behavior in a buffered system.

Fluorescence microscopy revealed densely distributed α‐syn droplets in the control group, displaying typical LLPS characteristics. Upon addition of 1‐ALPP, droplets appeared more regular and well‐defined. At a 1‐ALPP to α‐syn molar ratio of 1:1, droplet size decreased relative to the control group; although droplets comparable in size to those in the control group were still visible, their number was markedly reduced. When the molar ratio was increased to 3:1, both droplet size and number further decreased, demonstrating a dose‐dependent inhibitory effect (Figure 5K). These results indicate that 1‐ALPP effectively interferes with α‐syn LLPS, likely by disrupting the network of weak multivalent intermolecular interactions, thereby blocking droplet growth and fusion.

Given the complexity of the in vitro LLPS system, fluorescence microscopy alone is insufficient to quantitatively distinguish differences between treatment groups. Therefore, dynamic light scattering (DLS) was employed to quantitatively characterize the size distribution of LLPS droplets. The results showed that droplet sizes in all three groups were predominantly distributed within the 100–1000 nm logarithmic range (submicron to micron scale), with no significant signal in the 10–100 nm range, indicating the absence of small‐particle interference. The control group exhibited the largest droplet size among the three, with a characteristic peak at approximately 900 nm and a peak intensity of about 22. At a 1‐ALPP to α‐syn molar ratio of 1:1, the characteristic peak shifted to approximately 660 nm, with the peak intensity decreasing to about 18, reflecting a significant reduction in both droplet size and relative content compared to the control group. When the molar ratio was increased to 3:1, the characteristic peak further shifted to approximately 300 nm, with the peak intensity dropping to about 15 (Figure 5K), representing the smallest droplet size and lowest relative content among the three groups.

These results demonstrate that increasing concentrations of 1‐ALPP lead to a gradient decrease in both the characteristic droplet size and the relative content of LLPS droplets. Compared with the control group, both treatment groups exhibited significantly reduced droplet size and content, confirming that 1‐ALPP effectively inhibits the nucleation and growth of LLPS droplets in a concentration‐dependent manner.

### 3.8 E8 Exerts an Intergenerational Protective Effect on the PD Model via *hpl‐2*

Environmental exposures can sometimes induce intergenerational changes in gene expression. To investigate whether the neuroprotective effects of E8 could be inherited, we treated the A30P PD model (F1 generation) with the control bacteria OP50, despite its parental generation having been exposed to E8. The results demonstrated that the F1 generation still exhibited increased dendrite numbers (Figure 6A), suggesting the intergenerational transmission of the E8‐mediated effect. Notably, this neuroprotection persisted in the F2 generation, which had never been directly exposed to E8, but was lost by the F3 generation (Figure 6A), indicating a two‐generation transmission window. To exclude heritable suppression of the *dat‐1* promoter, we treated BY250 (P*dat‐1*::GFP) worms with E8 and quantified GFP intensity over three generations (F1–F3). No significant difference in fluorescence was observed between E8‐treated and control groups at any generation (Figure 6B), ruling out intergenerational repression of the *dat‐1* promoter. Thus, the intergenerational effects of E8 are unlikely to result from promoter silencing.

**FIGURE 6:**
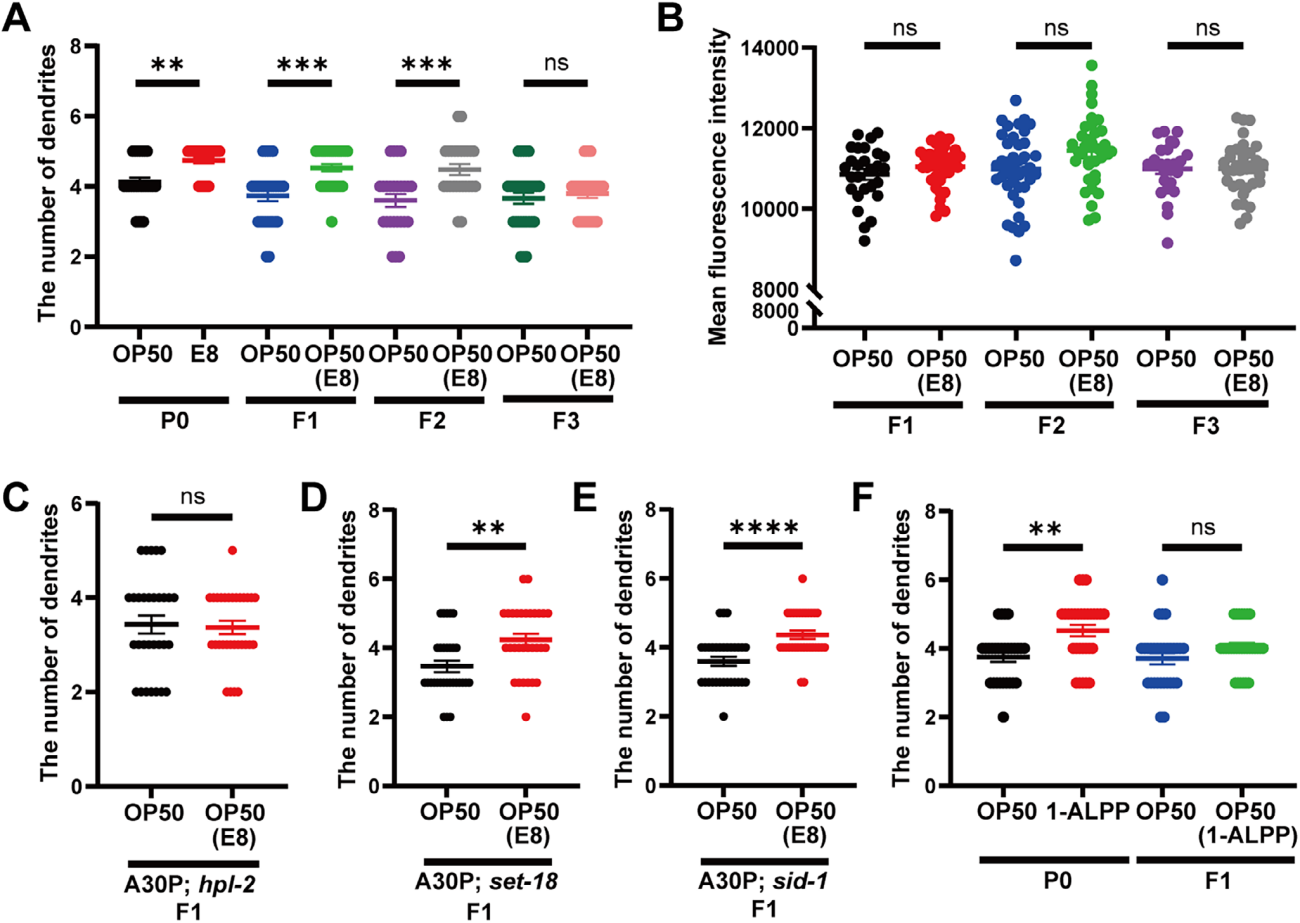
Intergenerational transmission of E8's effect on PD model. (A) Quantification of the dendrite numbers in PD models treated with OP50 or OP50 (E8). One‐way ANOVA followed by Tukey's post hoc test, *n* = 25–30. (B) Quantitative analysis of the fluorescence intensity of P*dat‐1*::GFP treated with OP50 or OP50 (E8). One‐way ANOVA followed by Tukey's post hoc test, *n* = 26–40. (C–E) Quantification of the dendrite numbers in PD models treated with OP50 or OP50 (E8) in *hpl‐2*, *set‐18*, *sid‐1* mutants or control groups. OP50 (E8) indicated that the worms were treated with OP50, while their parents were treated with E8. Two‐tailed unpaired Student's t‐test, *n* = 30. (F) Quantification of the dendrite numbers in PD models treated with OP50 or 1‐ALPP. One‐way ANOVA followed by Tukey's post hoc test, *n* = 28–30. Data were presented as mean ± SEM. ns, not significant (*p* > 0.05); ^**^ *p* < 0.01; ^***^ *p* < 0.001; ^****^ *p* < 0.0001.

To elucidate the underlying mechanism, we examined mutants of *hpl‐2*, which encodes an ortholog of heterochromatin protein 1 (HP1) [35], *set‐18*, which encodes a histone methyltransferase (HMT) [36], and *sid‐1*, a well‐characterized gene involved in RNA interference (RNAi) [37], previously implicated in intergenerational effects. Our data revealed that *hpl‐2* mutants, but not *set‐18 or sid‐1* mutants, blocked the intergenerational neuroprotective effect of E8 (Figure 6C–E), suggesting that *hpl‐2* is critical for E8‐mediated intergenerational inheritance. To determine whether the functional metabolite 1‐ALPP was responsible for this effect, we tested its intergenerational neuroprotective potential. However, 1‐ALPP did not confer neuroprotection in the F1 generation (Figure 6F), indicating that E8's intergenerational effect is independent of this metabolite.

## 4 Discussion

Parkinson's disease (PD) is a progressive, incurable neurodegenerative disorder, and the gut‐brain axis has become a focal point in PD pathogenesis research in recent years. A growing body of evidence has linked gut microbiota dysbiosis to α‐syn aggregation and dopaminergic neuron loss, with microbial metabolites proposed as potential therapeutic intermediaries. Our study identifies a novel *E. coli* strain with specific neuroprotective potential, which distinguishes it from previous gut microbial studies. The neuroprotective capacity of E8 is strain‐specific–‐other common *E. coli* subtypes do not replicate this effect, highlighting that functional diversity within a single bacterial species can drive distinct host neurophysiological responses.

A second core innovation lies in the identification of 1‐ALPP as the metabolite mediating E8's neuroprotection. While prior studies have identified microbial metabolites that either inhibit α‐syn fibrillogenesis or enhance autophagy, 1‐ALPP uniquely targets multiple sequential steps of α‐syn pathology, from liquid‐liquid phase separation to toxic aggregate formation, while also being non‐cytotoxic. This multi‐target action is distinct from single‐step inhibitors, which often fail to fully mitigate α‐syn‐mediated neurotoxicity.

Mechanistically, our finding that 1‐ALPP acts via the AMPK‐mTOR‐HLH‐30 autophagy pathway, while coordinating proteasomal clearance without affecting the lysosomal surveillance response, further underscores our study's novelty. Previous studies have established the importance of AMPK‐mTOR and HLH‐30/TFEB in PD, but none have linked these pathways to a specific gut bacterial metabolite. This connection bridges the gap between gut microbiota metabolism and conserved cellular degradation pathways, providing a molecular basis for how gut microbes regulate neuronal health in PD.

The *hpl‐2*‐dependent intergenerational neuroprotection by E8, independent of 1‐ALPP, represents another novel finding that extends beyond current understanding. Intergenerational effects of gut microbiota on neurodegeneration have rarely been reported, and our identification of *hpl‐2* as a critical mediator adds new insight into epigenetic regulation of gut‐brain axis effects in PD. This is distinct from prior intergenerational studies in *C. elegans*, which have not linked specific bacterial strains to neuroprotective epigenetic inheritance.

Collectively, our work advances PD research by shifting the focus from commonly studied probiotic genera to a unique *E. coli* strain and its metabolite, with a dual mechanism of action that offers both probiotic and metabolite‐based therapeutic potential. By demonstrating strain‐specific neuroprotection, multi‐target α‐syn regulation, and a novel intergenerational effect, our study provides new perspectives on gut‐brain crosstalk in PD, addressing unmet needs for targeted therapeutics that integrate microbial metabolism and neurodegenerative mechanisms.

The *C. elegans* model, while informative, has limitations in fully recapitulating human PD pathophysiology. Future studies should validate these findings in mammalian models, particularly in the context of α‐syn aggregation and motor dysfunction. Additionally, the clinical relevance of 1‐ALPP must be evaluated in human cohorts, including its pharmacokinetics and potential interactions with existing PD therapies.

## Author Contributions

Q.M., Z.X., C.X., S.J., X.M., W.F., L.J., S.J., N.W., L.Z., and L.L. conducted the experiments. Q.M., Z.X., C.X., S.J., and X.M. analyzed the data. W.X. wrote the paper, and all the authors contributed to manuscript writing. L.F. and W.X. proposed the concept, supervised the project, and revised the manuscript.

## Conflicts of Interest

The authors declare no conflicts of interest.

## Supporting information




**Supporting File**: advs78133‐sup‐0001‐SuppMat.docx.

## Data Availability

The data that support the findings of this study are available from the corresponding authors upon reasonable request.
