# Supplementary material for: Bacteria‐Derived 1‐Allylpiperazine Alleviates Symptoms of Parkinson's Disease Model by Promoting Autophagy
Source: Adv Sci (Weinh). 2026 Oct 8:e78133. Online ahead of print. doi: 10.1002/advs.78133 (PMC13650194; doi:10.1002/advs.78133)
Supplement: Supplementary file 1 [file ADVS-9999-e78133-s001.docx]

**Figure S1. The characteristics of the E8 strain.**

(A) The sequencing and comparison results of 16S rRNA of E8. (B) E8 whole genome sequencing phylogenetic tree of evolution. (C) Quantification of CFU for OP50 and E8. Two-tailed unpaired Student's t-test, n = 3 (biological replicate). (D-E) Statistical analysis of food preference of PD model and N2 for OP50 and E8. Two-tailed unpaired Student's t-test, n = 3-4 (biological replicate). (F) Statistical analysis of offspring numbers of PD model after OP50 or E8 treatment. Two-tailed unpaired Student's t-test, n = 2-3 (biological replicate). (G) Effect of E8 lysate obtained via ultrasonication on A30P. One-way ANOVA followed by Tukey’s post hoc test, n = 25-30. (H) Effect of E8 culture supernatant on A30P. Two-tailed unpaired Student's t-test, n = 28-30. (I) Effect of pasteurized E8 on A30P. One-way ANOVA followed by Tukey’s post hoc test, n = 38-43. (J) Effect of autoclaved E8 on A30P. One-way ANOVA followed by Tukey’s post hoc test, n = 25-27. (K) Statistical analysis of heat resistance of PD model after OP50 or E8 treatment. Two-tailed unpaired Student's t-test, n = 4 (biological replicate). (L) Statistical analysis of N2 heat resistance after OP50 or E8 treatment. Two-tailed unpaired Student's t-test, n = 4 (biological replicate). (M-N) Kaplan-Meier survival curves and mean lifespan of PD model or N2 treated with OP50 or E8. Two-tailed unpaired Student's t-test, n = 42-54. Data were presented as mean ± SEM. ns, not significant (*p* > 0.05); **p* < 0.05; ***p* < 0.01; *****p* < 0.0001.

**Figure S2. E8 ameliorates α-syn protein aggregation.**

(A) Representative fluorescence images and quantitative analysis of the normal dendrite numbers in the PD model after exposure to AA+6-OHDA and treatment with L-DOPA or E8. Arrows indicate the broken site of the dendrites of dopaminergic neurons. Scale bar = 20 μm, One-way ANOVA followed by Tukey’s post hoc test, n = 40. (B) Representative fluorescence images and quantitative analysis of α-syn protein aggregation in the dendrites of dopaminergic neurons. Scale bar = 20 μm, Two-tailed unpaired Student's t-test, n = 20-30. Data were presented as mean ± SEM. ns, not significant (*p* > 0.05); *****p* < 0.0001.
